# A Combination of Structural, Genetic, Phenotypic and Enzymatic Analyses Reveals the Importance of a Predicted Fucosyltransferase to Protein *O*-Glycosylation in the Bacteroidetes

**DOI:** 10.3390/biom11121795

**Published:** 2021-11-30

**Authors:** Markus B. Tomek, Bettina Janesch, Matthias L. Braun, Manfred Taschner, Rudolf Figl, Clemens Grünwald-Gruber, Michael J. Coyne, Markus Blaukopf, Friedrich Altmann, Paul Kosma, Hanspeter Kählig, Laurie E. Comstock, Christina Schäffer

**Affiliations:** 1 *NanoGlycobiology* Unit, Institute of Biologically Inspired Materials, Department of NanoBiotechnology, Universität für Bodenkultur Wien, Muthgasse 11, A-1190 Vienna, Austria; markus.tomek@boku.ac.at (M.B.T.); bettina.janesch@gmx.at (B.J.); math.b@gmx.at (M.L.B.); taschner@lifetaq.com (M.T.); 2 Institute of Biochemistry, Department of Chemistry, Universität für Bodenkultur Wien, Muthgasse 18, A-1190 Vienna, Austria; rudolf.figl@boku.ac.at (R.F.); clemens.gruber@boku.ac.at (C.G.-G.); friedrich.altmann@boku.ac.at (F.A.); 3 Department of Microbiology and the Duchossois Family Institute, University of Chicago, KCBD, 900 E. 57th Street, Chicago, IL 60637, USA; mcoyne@uchicago.edu (M.J.C.); lecomstock@uchicago.edu (L.E.C.); 4 Institute of Organic Chemistry, Department of Chemistry, Universität für Bodenkultur Wien, Muthgasse 18, A-1190 Vienna, Austria; markus.blaukopf@boku.ac.at (M.B.); paul.kosma@boku.ac.at (P.K.); 5 Department of Organic Chemistry, Faculty of Chemistry, University of Vienna, Währinger Strasse 38, A-1090 Vienna, Austria; hanspeter.kaehlig@univie.ac.at

**Keywords:** *Bacteroides fragilis*, *O*-glycan structure, glycosyltransferase, *Pedobacter heparinus*, *Tannerella forsythia*, l-fucose, relaxed acceptor specificity

## Abstract

Diverse members of the Bacteroidetes phylum have general protein *O*-glycosylation systems that are essential for processes such as host colonization and pathogenesis. Here, we analyzed the function of a putative fucosyltransferase (FucT) family that is widely encoded in Bacteroidetes protein *O*-glycosylation genetic loci. We studied the FucT orthologs of three Bacteroidetes species—*Tannerella forsythia*, *Bacteroides fragilis*, and *Pedobacter heparinus*. To identify the linkage created by the FucT of *B. fragilis*, we elucidated the full structure of its nine-sugar *O*-glycan and found that l-fucose is linked β1,4 to glucose. Of the two fucose residues in the *T. forsythia* *O*-glycan, the fucose linked to the reducing-end galactose was shown by mutational analysis to be l-fucose. Despite the transfer of l-fucose to distinct hexose sugars in the *B. fragilis* and *T. forsythia* *O*-glycans, the FucT orthologs from *B. fragilis*, *T. forsythia*, and *P. heparinus* each cross-complement the *B. fragilis* Δ*BF4306* and *T. forsythia* Δ*Tanf_01305* FucT mutants. In vitro enzymatic analyses showed relaxed acceptor specificity of the three enzymes, transferring l-fucose to various *p*NP-α-hexoses. Further, glycan structural analysis together with fucosidase assays indicated that the *T. forsythia* FucT links l-fucose α1,6 to galactose. Given the biological importance of fucosylated carbohydrates, these FucTs are promising candidates for synthetic glycobiology.

## 1. Introduction

Bacteroidetes is a phylum of Gram-negative bacteria that colonize diverse ecological niches. Within this phylum are members of the order Bacteroidales, which include abundant anaerobic gut symbionts such as *Bacteroides* species that provide benefits to their host [1,2], as well as pathogenic anaerobic species such as the periodontal pathogens *Tannerella forsythia* and *Porphyromonas gingivalis* [3]. Flavobacteriales and Sphingobacteriales are other orders of this phylum that are generally aerobes or facultative anaerobes and typically colonize diverse environmental and non-mammalian host ecosystems. Members of this phylum have a tremendous capability to degrade high molecular-weight polysaccharides using dedicated polysaccharide utilization loci, many of which are shared between distantly related members [4].

One outstanding feature of many Bacteroidales species is their synthesis of numerous capsular polysaccharides (CPS) and glycoproteins. For example, the gut symbiont *B. fragilis* NCTC 9343 produces eight CPSs that are subject to phase variation, each synthesized by the products of separate operons [5,6]. This strain also has a general protein *O*-glycosylation system that targets hundreds of proteins of diverse biological functions at a conserved (D)(S/T)(A/I/L/V/M/T/S/C/G/F) motif [7,8]. A genetic locus termed *lfg* (locus for glycosylation) is necessary for protein glycosylation with the full-length oligosaccharide in *B. fragilis*, and *lfg*-like regions are present in the genomes of *Bacteroides* species. In all *Bacteroides* species analyzed, the *lfg* region begins with *metG* and terminates with an ortholog of the *B. fragilis* NCTC 9343 gene *BF4306* (alternate designation *BF9343_4192*), predicted to encode a fucosyltransferase [7]. The transcriptional linkage of the *lfg* region to *metG*, a gene required for translation, implies the importance of protein glycosylation in these bacteria [7]. *T. forsythia* is a periodontal pathogen, also of the Bacteroidales order, which synthesizes a prominent glycosylated cell surface (S-) layer comprised of the glycosylated proteins TfsA and TfsB. In addition, several other proteins of *T. forsythia* are glycosylated using this general protein *O*-glycosylation system [9,10]. The mature *O*-glycan is pivotal to the establishment of *T. forsythia* in the oral biofilm community (dental plaque) [11], its recognition by the immune system in a macrophage cell culture model [12], and the modulation of dendritic cell effector functions upon infection [10]. Cross-glycosylation analyses between *T. forsythia* and *B. fragilis* confirmed that the conserved three-amino acid glycosylation motif identified in *B. fragilis* is also the site of protein glycosylation in *T. forsythia* [7,8].

The presence of fucose (Fuc) residues in carbohydrate structures is crucial for many biological and pathological processes in both eukaryotic and prokaryotic organisms. Fucosylated bacterial oligosaccharides have been suggested to be involved in molecular mimicry, adhesion, colonization, and modulation of immune responses [13,14,15]. Previous studies have shown that both the CPSs and *O*-glycans of Bacteroidales contain Fuc [7,16]. A *B. fragilis* Δ*gmd-fcl*Δ*fkp* mutant [16], with deletions of genes involved in both the de novo and salvage pathways for synthesis of GDP-l-Fuc [15], the precursor for incorporation of l-Fuc into bacterial glycans and polysaccharides, is rapidly outcompeted by wild-type bacteria in a mouse model of intestinal colonization [16,17]. Structural elucidation of the *O*-glycan of *T. forsythia* revealed that this critical virulence compound also contains Fuc [9,10].

In bacteria, fucosyltransferases (FucTs) transfer Fuc from either GDP-l-Fuc or TDP-d-Fuc into a growing undecaprenol-P linked oligosaccharide at the cytoplasmic face of the inner membrane. Several putative bacterial FucT genes have been identified to date, but only a few are functionally characterized (reviewed previously [14,18]). The best-studied bacterial enzymes are the α1,2-, α1,3-, and α1,3/4-l-FucTs from *Helicobacter pylori* responsible for the last steps in the synthesis of Lewis blood antigen structures [19]. Structural data for bacterial FucTs are available for the *H. pylori* α1,3-l-FucT [20] and the *Bradyrhizobium* sp. WM9 α1,6-l-FucT NodZ involved in chitooligosaccharide Nod-factor biosynthesis [21], highlighting the limited characterization of prokaryotic FucTs.

A predicted FucT, Tanf_01305 (named GtfE [10]) is involved in the biosynthesis of the complex *O*-linked decasaccharide of *T. forsythia* that modifies several of the bacterium’s proteins [8,9]. Analysis of the *O*-glycan of a *Tanf_01305* deletion mutant showed that the glycan was truncated and lacked the fucose residue branching from the reducing end galactose [10]. Analysis of the deletion mutant of the FucT ortholog in *B. fragilis* NCTC9343 (Δ*BF4306*) showed that only the first two sugars are added to the glycan, despite the fact that mass spectrometry analysis revealed a deoxyhexose (i.e., the predicted l-Fuc) to be the fourth sugar of the glycan [7,22]. The same phenotype results in the *gmd-fcl/fkp* mutant, which is unable to synthesize GDP-l-fucose, the precursor for addition of l-Fuc into the glycan. Therefore, the data support that the *B. fragilis* FucT ortholog is an l-Fuc transferase.

In this study, we sought to study the *BF4306* gene product of *B. fragilis*, determine the linkage it creates in the *O*-glycan structure, and compare it to the predicted FucT of *T. forsythia* (Tanf_01305) and the bioinformatically predicted FucT of the distantly related Bacteroidetes species *Pedobacter heparinus* (Phep_4048). We elucidated the *B. fragilis* NCTC 9343 *O*-glycan structure by 1D and 2D NMR spectroscopy and identified the Fuc linkage. In addition, we performed cross-complementation experiments using the FucT from *T. forsythia* ATCC 43037, *B. fragilis* NCTC 9343 and *P. heparinus* DSM 2366 accompanied by mass spectrometry together with an in vitro enzyme assay to reveal relaxed acceptor specificity of these novel FucTs.

## 2. Materials and Methods

### 2.1. Construction of a Cladogram of Bacteroidetes Fucosyltransferases

The proteomes of each of thirty-five Bacteroidetes genomes were compiled into a custom blast database using makeblastdb from the BLAST+ suite (version 2.10.0) (National Library of Medicine (US), National Center for Biotechnology Information, Bethesda, MD, USA). Two predicted FucT orthologs (BF4306, *B. fragilis* NCTC 9343 NC_003228: 5112881..5113649; Tanf_01305, *T. forsythia* ATCC 43037 NZ_JUET01000030: 51799..52566) were used to query this database using blastp, and the best hits from each genome to each query by bitscore were retained. In all cases, the best target protein sequence found was the same for both queries. MEGA X (version 10.2.2) (Molecular Evolutionary Genetics Analysis; Megasoftware at www.megasoftware.net) [23] was used to generate a Clustal W alignment of these 35 FucT proteins and to generate the maximum likelihood phylogenetic tree using 250 bootstrap replicates [24] and JTT model [25] for amino acid substitutions.

### 2.2. Bacterial Strains and Cultivation Conditions

*T. forsythia* ATCC 43037 (American Type Culture Collection, Manassas, VA, USA), characterized *T. forsythia* mutants, and *B. fragilis* NCTC 9343 (National Collection of Type Cultures, Salisbury, UK) were grown anaerobically in Brain Heart Infusion (BHI) medium as described previously [10]. *P. heparinus* DSM 2366 (German Collection of Microorganisms and Cell Cultures, Braunschweig, Germany) was grown in peptone (5 g/L)-meat extract (3 g/L) medium at 28 °C under aerobic conditions. The following antibiotics were added when appropriate: 50 µg/mL gentamicin, 5 µg/mL erythromycin or 10 µg/mL chloramphenicol. *Escherichia coli* strains were grown under standard conditions in Luria Bertani medium (LB; Sigma-Aldrich, Vienna, Austria) containing 100 μg/mL ampicillin and 50 µg/mL kanamycin, when appropriate. All bacterial strains and plasmids used in this study are listed in Table 1.

### 2.3. General Methods

Genomic DNA was extracted according to a published protocol [32]. Plasmid DNA was isolated with the GeneJET Plasmid Miniprep Kit (Thermo Fisher Scientific, Vienna, Austria). Oligonucleotides (Thermo Fisher Scientific) used in this study are listed in Table S1. PCR amplification was performed with Phusion High-Fidelity DNA polymerase (Thermo Fisher Scientific) according to the manufacturer’s instructions. The GeneJET Gel Extraction Kit (Thermo Fisher Scientific) was used to purify DNA fragments and restriction enzyme (Thermo Fisher Scientific)-digested plasmids. Transformation of chemically competent *E. coli* DH5α and BL21(DE3) cells (Thermo Fisher Scientific) was performed according to the manufacturer’s protocol. Transformants were screened by PCR using the REDTaq ReadyMix (Sigma-Aldrich, Vienna, Austria) and confirmed by sequencing (Microsynth, Vienna, Austria).

SDS-PAGE was performed according to a standard protocol [33] in a Mini-Protean II electrophoresis apparatus (Bio-Rad, Vienna, Austria). Carbohydrates were visualized with ProQ-Emerald dye [34]. Protein bands were visualized with colloidal Coomassie Brilliant Blue R-250 (Serva, Heidelberg, Germany) or were transferred onto a nitrocellulose or polyvinylidene difluoride (PVDF) membrane (Bio-Rad) for Western-blot analysis. Polyclonal rabbit antisera raised against the recombinant *T. forsythia* S-layer proteins TfsA (α-TfsA) and TfsB (α-TfsB) [35] were used as primary antibodies in combination with a monoclonal goat α-rabbit IgG secondary antibody labeled with IRDye 800CW (LI-COR Biosciences, Lincoln, NE, USA). *B. fragilis* cell lysates were probed with antiserum to the unglycosylated His-tagged BF2494 protein [36] followed by a monoclonal goat α-rabbit IgG secondary antibody as above. Bands were visualized at 800 nm using an Odyssey Infrared Imaging System (LI-COR Biosciences). Protein concentrations were determined using the Bradford Assay Kit (Bio-Rad) and carbohydrates in fractions after column purification were determined using the orcinol assay [37].

### 2.4. Preparation of Bacteroides fragilis Glycopeptides for Glycan Structure Elucidation

To obtain sufficient material for *B. fragilis* *O*-glycan structure elucidation by NMR spectroscopy, we took advantage of the bacterium’s general protein *O*-glycosylation system where the same *O*-glycan is added to numerous extracytoplasmic proteins [7]. To obtain glycopeptides, 5-g batches of *B. fragilis* wild-type biomass (wet pellet) were digested with 100 mg of Pronase E (Sigma-Aldrich) in 150 mM Tris-HCl buffer, pH 7.8, containing 1 mM CaCl_2_ and 0.02% NaN_3_, at 37 °C for 24 h [38]. These digests were pre-purified using a Dowex 50WX2 cation-exchange resin (H^+^-form; Sigma-Aldrich) according to the manufacturer’s instruction, followed by size exclusion chromatography on a Sephadex G50 superfine column (120 × 2.5 cm) with 1% acetic acid as eluent. Elution was monitored by measuring the absorbance at 235 nm and fractions of 10 mL were collected. Final isolation and fractionation of glycopeptides from a prominent carbohydrate-positive pool was performed by preparative porous graphitized carbon (PGC) HPLC, employing a Hypercarb column (150 × 3 mm, 5 µm particle size; Thermo Fisher Scientific) with a gradient of 1% to 80% solvent B in solvent A over 60 min at flow rate of 0.5 mL/min (solvent A: 80 mM ammonium formate, pH 3.0; solvent B: 20% solvent A, 80% acetonitrile (ACN)) and a fraction size of 0.25 mL [9].

To facilitate NMR analysis of the protein-linked reducing-end sugar of the *O*-glycan, the glycopeptide preparation procedure was also performed for the *B. fragilis* Δ*BF4306* mutant, which produces a heavily truncated *O*-glycan comprising only two sugar residues [36]. PGC-LC-ESI-MS screening of *B. fragilis* Δ*BF4306* glycopeptides was performed as described in Section 2.5 for the analysis of β-eliminated *O*-glycans.

### 2.5. β-Elimination of Glycans and Mass Spectrometry Analysis

*O*-glycans were released from the purified pool of *B. fragilis* wild-type glycopeptides by reductive β-elimination with 1 M NaBH_4_ in 0.5 M NaOH at 50 °C overnight [39], followed by purification of the reduced *O*-glycans by preparative PGC-HPLC as described above. *O*-glycans from *T. forsythia* wild-type and cross-complemented *T. forsythia* Δ*Tanf_01305* strains were released from glycoproteins after separation by SDS-PAGE by in-gel reductive β-elimination [9,10]. After enrichment and clean-up via PGC SPE cartridges (10 mg HyperSep Hypercarb, Thermo Scientific) using the same solvents and elution strength as in the subsequent chromatography, the glycan mixtures were analyzed on a PCG-Hypercarb column (100 × 0.32 mm, 5 µm particle size; Thermo Fisher Scientific) with a gradient of 1% to 80% solvent B in solvent A over 15 min at flow rate of 6 µL/min (solvent A and B as in Section 2.4 above), using a Dionex Ultimate 3000 system directly linked to an ion trap instrument (amaZon speed ETD, Bruker, Germany) equipped with the standard ESI source in positive-ion, data-dependent acquisition (DDA) mode (performing MS^2^ on signals based on their intensity and LC elution, at 30% collision energy using CID with helium gas). MS-scans were recorded over an *m/z* range of 450–1650; the ICC target was set to 100,000 and maximum accumulation time to 200 ms. The top 10 highest peaks were selected for fragmentation with an absolute intensity threshold above 50,000. Instrument calibration was performed using ESI Tuning Mix (Agilent Technologies, Vienna, Austria) as per the manufacturer’s recommendations.

Data were evaluated manually using the DataAnalysis 4.0 software (Bruker) and Glycoworkbench 2.1 build 146 [40].

### 2.6. NMR Spectroscopy for Structure Analysis of the Bacteroides fragilis O-Glycan

NMR spectra were recorded on a Bruker AV III HD 700 MHz NMR spectrometer (Bruker BioSpin, Rheinstetten, Germany), equipped with a quadruple (^1^H, ^13^C, ^15^N, ^19^F) inverse helium-cooled cryo-probe, operating at 700.40 MHz for ^1^H and 176.12 MHz for ^13^C, respectively. A 500-µg sample of purified, β-eliminated *B. fragilis* wild-type glycan dissolved in 300 µL of D_2_O and transferred into a Shigemi tube, as well as three individual fractions of *B. fragilis* Δ*BF4306* glycopeptides dissolved in 600 µL of D_2_O and transferred into standard 5-mm NMR tubes, were measured at a temperature of 25 °C. The spectra were referenced for ^1^H to the signal of the methyl groups of DSS (δ = 0 ppm), and for ^13^C on a unified scale relative to ^1^H using the Ξ value for DSS [41]. The following experiments were performed using pulse sequences as supplied by the manufacturer: ^1^H NMR with and without suppression of the HDO signal using presaturation or a diffusion filter (diffusion delay of 100 ms), ^13^C DEPTq-135, 2D DQF-COSY, 2D TOCSY (100 ms MLEV17 spin-lock), 2D NOESY (500 ms mixing time), 2D HSQC with and without ^13^C decoupling (GARP), and 2D HMBC. In addition, 1D selective TOCSY experiments were performed for high resolution spectra of the sugar units using a selective pulsed-field gradient spin echo sequence (80 ms 180° Gaussian pulse, 100 to 300 ms MLEV17 spin-lock).

Processing and detailed analysis of the spectra were performed within the TopSpin software (Bruker BioSpin). The spin coupling network was elucidated by spin simulations using DAISY within the TopSpin software, by fitting the calculated spectra mainly to the 1D TOCSY traces or partly to the normal ^1^H NMR spectrum.

### 2.7. Cross-Complementation in Tannerella forsythia

A cross-complementation gene cassette was constructed to replace the native *Tanf_01305* gene with the homologous genes *BF4306* from *B. fragilis* and *Phep_4048* from *P. heparinus*, respectively. A detailed description of the cloning procedure and the transformation of vectors into *T. forsythia* is published elsewhere [30]. Briefly, the native *Tanf_01305* upstream region was amplified with primers 490 and JB_6. The *BF4306* gene was amplified using primer pair JB_7/JB_23 containing the restriction sites KpnI and SacI. Next, this fragment was added to the upstream region by overlap-extension (OE) PCR and subcloned into the blunt-end cloning vector pJET1.2. The chloramphenicol (*cat*) resistance gene was amplified from pJET1.2/Δ*Tanf_01245*^+^ using primers JB_24 (KpnI) and JB_10 [31] (Table S1), and cloned to the native down-stream homology region, JB_11/JB_19 (SacI), by OE-PCR. Via the introduced restriction sites KpnI and SacI, the combined *cat* gene and the down-stream homology region were inserted, creating the final cross-complementation cassette pJET1.2/Δ*Tanf_01305*^+*BF4306*^.

Analogously, the cross-complementation cassette pJET1.2/Δ*Tanf_01305*^+*Phep_4048*^ for the integration of the homologous gene *Phep_4048* from *P. heparinus* was created. The sole exception was the use of primers 490/JB_13 for the amplification of the upstream homology region and JB_14/JB_25 (KpnI, SacI) for the amplification of the *Phep_4048* gene. Clones were selected on chloramphenicol-containing BHI plates and tested for correct integration on the genomic level after transformation into electrocompetent *T. forsythia* Δ*Tanf_01305* cells [31] (Figure S1).

### 2.8. Cross-Complementation in Bacteroides fragilis

Genes *Tanf_01305* and *Phep_4048* were cloned into an expression vector for complementation studies in *B. fragilis* ΔBF4306. The *Phep_4048* gene was PCR-amplified using *P. heparinus* DSM 2366 genomic DNA as template, with primers Phep_4048_F/Phep_4048_R, which included a BamHI site (Table S1), and this product was inserted into BamHI-linearized pCMF118 [22], creating pMT2. Analogously, the *Tanf_01305* gene was PCR-amplified using *T. forsythia* ATCC 43037 genomic DNA as template with primers Tanf_01305_F/Tanf_01305_R, which included BamHI sites, and was inserted into BamHI-linearized pCMF118 [22], creating pMT21.

These plasmids were transferred from *E. coli* DH5α into *B. fragilis* Δ*BF4306* by conjugation using helper plasmid RK231. Transconjugants were selected using erythromycin and gentamycin.

### 2.9. Construction of a T. forsythia GDP-l-Fucose Synthase Deletion Mutant

A knock-out vector was constructed to exchange the GDP-l-fucose synthase gene of *T. forsythia* (*Tanf_07535, fcl*) in frame with the erythromycin resistance gene, *erm.* The plasmid contains regions of approximately 1-kbp upstream and downstream of *Tanf_07535*, flanking *erm*. Primer pairs 486/487 and 488/489, respectively, were used to amplify the up- and down-stream homology regions from genomic DNA of *T. forsythia*. The *erm* gene (805 bp, without the promotor region) was amplified from pJET/TF0955ko [30] using primers 460 and 461 (Table S1). This gene cassette was blunt-end cloned into the cloning vector pJET1.2, creating the final knock-out vector pJET1.2/Δ*Tanf_07535*. Transconjugants selected on plates containing erythromycin and gentamycin were further confirmed by screening PCR (Figure S2). The deletion mutant carries an *ermF* marker in place of the *fcl* gene and, accordingly, was named Δ*Tanf_07535*::*ermF*.

### 2.10. Cloning of Fucosyltransferase Genes

The fucosyltransferase genes *Tanf_01305* (KKY62509.1), *BF4306* (CAH09973.1), and *Phep_4048* (ACU06239.1) were PCR amplified from genomic DNA using primers 516/517, JB_174/JB_175, and JB_172/JB_173, respectively. The amplification products were digested with EcoRI/HindIII (*Tanf_01305*, *BF4306*) and EcoRI/BamHI (*Phep_4048*) and inserted into the linearized vector pMAL_c2E digested with the same restriction enzymes (amino acid sequences of the maltose binding protein (MBP)-fusion proteins can be found in Figure S3). These plasmids, pMAL_c2E/Tanf_01305, pMAL_c2E/BF4306, and pMAL_c2E/Phep_4048, were transformed into *E. coli* BL21(DE3) cells for FucT expression. All constructs were verified by sequencing.

### 2.11. Expression and Purification of Recombinant Fucosyltransferases

Overnight cultures of *E. coli* BL21(DE3) harboring each the FucT encoding plasmids were inoculated into 400 mL of 2× LB medium with ampicillin and cells were grown at 37 °C and 200 rpm until an OD_600_ of 0.4 to 0.6 was reached. The cultures were shifted to 30 °C and expression was induced by addition of 0.5 mM IPTG. After incubation for 4 h, cells were harvested by centrifugation and cell pellets were stored at −20 °C. Cell pellets were thawed and resuspended in 20 mM Tris-HCl, pH 7.5 (15 mL buffer per g of wet cell pellet) in the presence of a protease inhibitor cocktail (cOmplete, Roche, Vienna, Austria). Cells were lysed by sonication and cell debris was removed by centrifugation (20,000× *g*, 20 min, 4 °C). Supernatants containing the soluble recombinant proteins were purified via an amylose resin (New England Biolabs, Vienna, Austria; running buffer: 20 mM Tris-HCl, pH 5.0, 200 mM NaCl; elution buffer: 20 mM Tris-HCl, pH 7.5, 200 mM NaCl, 10 mM maltose). Fractions were monitored and those containing the enzyme, based on SDS-PAGE analysis, were pooled. rTfFuc1 was expressed and purified as described previously [42].

### 2.12. In Vitro Fucosyltransferase Activity Assays Using pNP-Sugar Substrates

The activity of the recombinant FucTs was determined by using 4-nitrophenyl (*p*NP)-α-d-Gal, *p*NP-α-d-Glc, *p*NP-α-d-Man, *p*NP-β-d-Xyl, *p*NP-α-d-GlcA, and *p*NP-β-d-GlcNAc as substrates (Sigma-Aldrich). The standard assay conditions at 37 °C were: 5 µL (~15–20 µg) of rFucT enzyme, 8 mM *p*NP-sugar substrate, 20 mM Tris-HCl, pH 7.4, 5 mM MgCl_2_, 5 mM MnCl_2_, and 2 mM GDP-L-Fuc (Sigma-Aldrich). After overnight incubation, reactions were terminated by the addition of 1:1 (*v*/*v*) stop solution (80% ACN). Samples were analyzed by thin-layer chromatography on silica plates (TLC Silica gel 60 F_254_, Merck Millipore, Vienna, Austria) with ACN:H_2_O (17:2) as running solvent. Separated spots were detected on the dried plates under UV-light at 254 nm.

For product analysis by ESI-MS, reactions were terminated by heating at 60 °C for 10 min followed by centrifugation to remove precipitated proteins. The supernatant was injected in the iontrap mass analyzer; instrument parameters were as described in Section 2.5, except for the recorded *m*/*z* range, which was 200–1650.

### 2.13. Preparation of pNP-α-d-Gal-Fuc as a Fucosidase Substrate

The preparation of *p*NP-α-d-Gal-Fuc is described in Supplementary Materials.

## 3. Results

### 3.1. Presence of FucT Orthologs in Diverse Bacteroidetes Genomes

**Figure 1:**
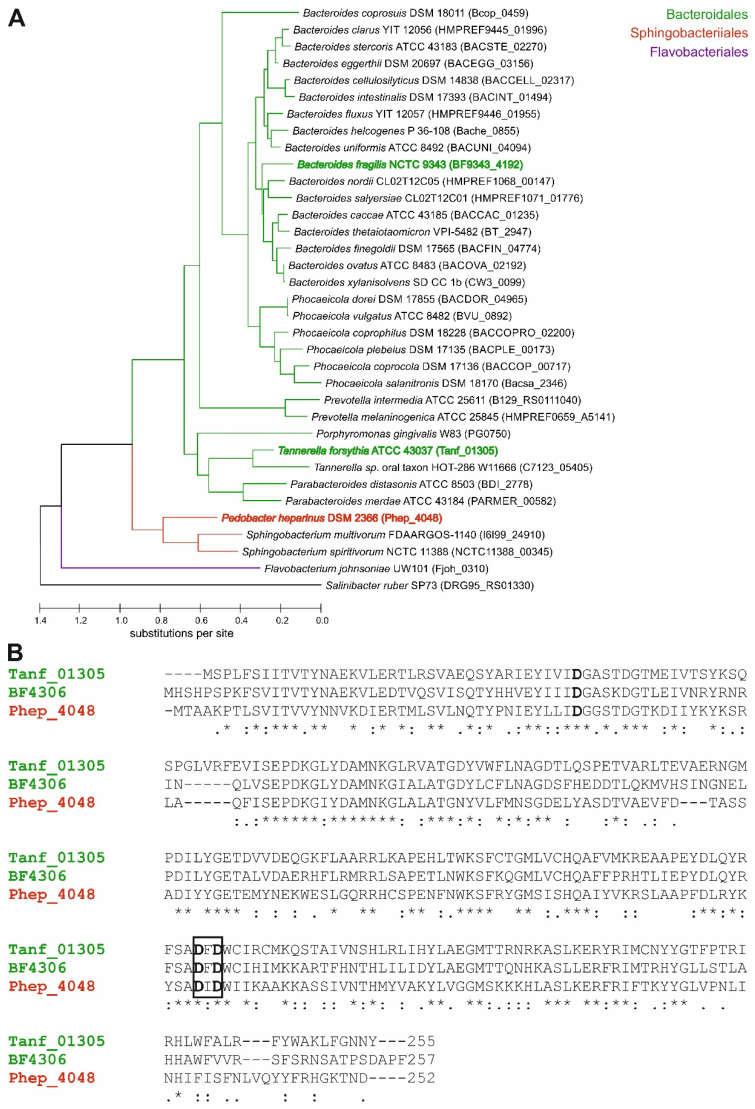
(**A**) Cladogram of the FucT orthologs in diverse species of the Bacteroidetes phylum. The maximum likelihood tree with the highest log likelihood (−9554.64) is shown, with initial trees generated using the Neighbor-Join and BioNJ methods applied to a JTT matrix-based model [25] of pairwise distances. The branch lengths of the tree indicate the number of substitutions per site (35 amino acid sequences comprising 359 positions). The tree was produced using MEGA X [23]. (**B**) Amino acid sequence alignment of *T. forsythia* Tanf_01305 (KKY62509.1), *B. fragilis* BF4306 (CAH09973.1), and *P. heparinus* Phep_4048 (ACU06239.1) illustrates the sequence similarity for compared sequences. The conserved DXD-motif typical of glycosyltransferases is indicated within a black box, and predicted metal binding sites are written in boldface. Amino acids identical in all three proteins are indicated with *. The alignment was calculated with the software Clustal Omega (www.ebi.ac.uk/Tools/msa/clustalo; accessed on 1 July 2021).

Mutational analysis of BF4306 and Tanf_01305 suggested that these orthologs are FucTs. In the *O*-glycan biosynthesis regions of all *Bacteroides* species analyzed, and in *T. forsythia*, this gene is conserved and is the terminal gene of these biosynthesis loci. These predicted FucTs are from the broad GT2 family of glycosyltransferases with a predicted GT-A type structural fold [10]. To determine how prevalent these FucT orthologs are in Bacteroidetes species, we searched the genomes of 35 diverse Bacteroidetes strains for orthologs. Despite the fact that the *B. fragilis* and *T. forsythia* FucT are only 71% similar to each other, separate Blastp searches using the *B. fragilis* and *T. forsythia* FucT proteins retrieved the same ortholog in each genome. The cladogram (Figure 1A) illustrates that the relationships among these FucT orthologs seemed to parallel species phylogeny. Glycosyltransferase-encoding genes of Bacteroidales species are also commonly found in non-conserved segments of the genome, like CPS biosynthesis loci, so this phylogenic distribution was unexpected.

Alignment of the *B. fragilis*, *T. forsythia*, and distantly related *P. heparinus* FucT orthologs show that they all contain a DXD motif typical of glycosyltransferases [43] (Figure 1B).

### 3.2. The Bacteroides fragilis O-Glycan Is a Complex Nonasaccharide Containing Fucose Linked α1,4 to Glucose

The analysis of Figure 1 illustrates the conservation of this FucT in Bacteroidetes species, suggesting its importance in *O*-glycosylation in this phylum. Our previous analysis of the *B. fragilis* protein *O*-glycan showed that it is composed of nine monosaccharides categorized broadly (i.e., hexose, deoxyhexose, etc.) [7,22]. By analysis of mutants unable to synthesize GDP-l-fucose, we showed that l-Fuc is a component of the outer glycan of *B. fragilis* and is also present in the *O*-linked glycans of diverse other Bacteroidetes species [7,16]. To unambiguously determine the position and linkage of the fucose moiety in the *B. fragilis* *O*-glycan and the various linkages between residues, we sought to elucidate the complete structure of this glycan. Reductive β-elimination was employed to release the *O*-glycan from purified *B. fragilis* glycopeptides. Subsequent PGC-ESI-MS analysis of the derived glycan revealed a molecule with a monoisotopic value of *m*/*z* = 1571.5 Da, in agreement with previous data from our laboratory and corresponding to a nonasaccharide [22].

The ^1^H NMR of the *B. fragilis* *O*-glycan preparation showed a typical carbohydrate spectrum with anomeric and core signals in the narrow chemical shift range between 3.2 and 5.3 ppm (Figure S4). Within this region, a sharp singlet at 3.44 ppm hinted a methoxy group and signals in the aliphatic region were indicative of an acetyl group (~2.0 ppm) and deoxy sugars (1.2 ppm).

**Figure 2:**
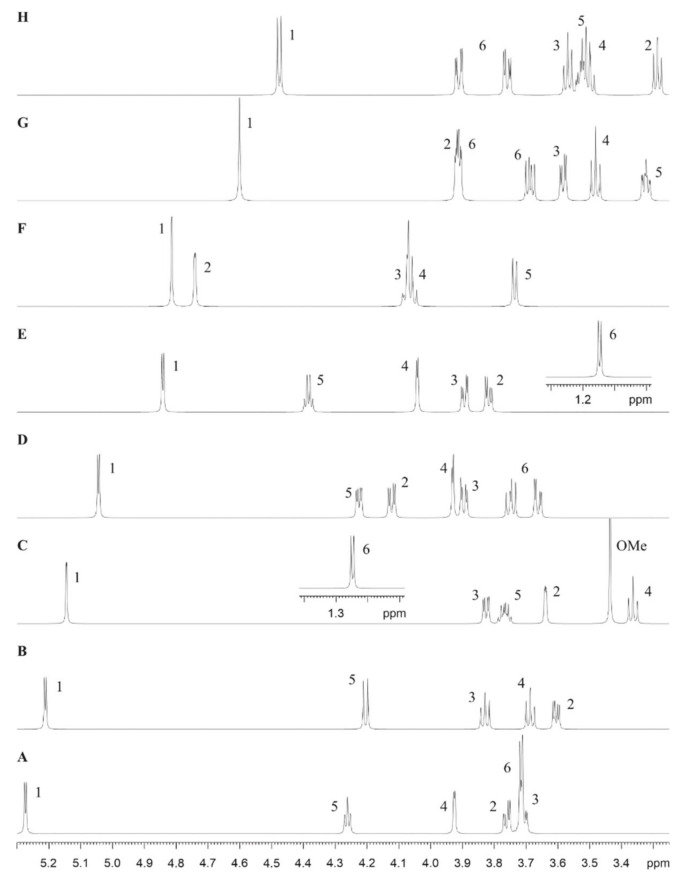
Simulated ^1^H NMR spectra of the sugar units (**A**–**H**) of the *B. fragilis* wild-type *O*-glycan released from glycopeptides. The traces **A**–**H** show the individual spin systems together with the assignment of the sugar protons. OMe, O-methyl.

**Figure 3:**
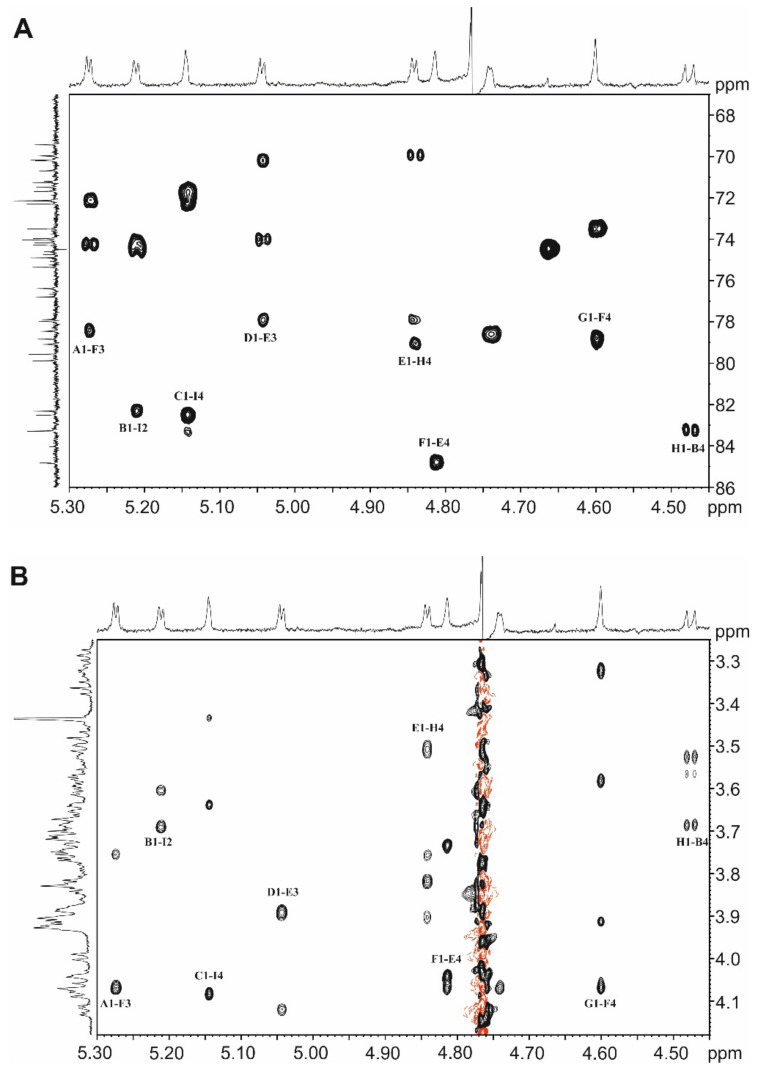
(**A**) Region of the 2D-HMBC spectrum and (**B**) region of the 2D-NOESY spectrum of the *B. fragilis* wild-type *O*-glycan released from glycopeptides by reductive β-elimination, showing the inter-glycosidic cross-peaks. (**A**) Long-range cross-peaks over three bonds between the anomeric protons of residues **A**–**H** to the carbon at the linkage position of the next following sugar and (**B**) NOE cross-peaks between the anomeric protons of residues **A**–**H** to the proton at the linkage position of the next following sugar.

The anomeric region in a 2D HSQC spectrum showed eight signals that gave a cross-peak at a ^13^C chemical shift between 100 and 105 ppm, characteristic of anomeric carbons (Figure S5). To identify the individual monosaccharides, the anomeric signals were chosen as starting point, providing a unique reporter for each monomer (**A**–**H**) due to clear separation. The anomeric proton of **A** had a splitting of 4 Hz; the cross-peak in the DQF-COSY to proton 2 showed a significant larger coupling, therefore, not representing a mannose-type sugar. The 2D TOCSY gave a cross-peak with a very narrow shape for proton 4, assigning sugar **A** as α-Gal*p*. To verify this sugar, the derived chemical shifts and the estimated coupling constants were used as input for a spin simulation and optimized by fitting to the experimental spectra, essentially to a 1D TOCSY trace. Figure 2 shows the calculated spectra together with the assignment of sugar **A** as well as of all following residues. For sugar **B**, besides the small splitting of 4 Hz for the anomeric proton, only large coupling constants can be detected (Figure 2). The proton spin system ends at position 5 with a doublet a 4.205 ppm. The 2D HMBC spectrum showed a cross-peak at this proton frequency to the carbon region related to carboxylic groups, thus identifying **B** as α-GlcA. The 2D TOCSY trace for the anomeric signal of residue **C** showed a cross-peak in the aliphatic region indicating a 6-deoxy sugar. Together with only small *J* couplings for H-2, **C** is Rha*p* (Figure 2). The anomeric configuration was deduced from the size of the proton-carbon coupling constant in a 2D HSQC experiment without ^13^C decoupling during acquisition. With an experimental value of 168.4 Hz, **C** refers to α-Rha*p*. In addition, this building block was methylated at position 2, indicated by HMBC cross-peaks from the CH_3_ protons at 3.44 ppm to the appropriate carbon C-2 at 83.30 ppm, and from the methyl carbon at 61.09 ppm to H-2 at 3.64 ppm (Figure 3A). For building block **D**, the coupling pattern of the 2D DQF-COSY and 2D TOCSY cross-peaks with small *J* values between 1 and 2, large *J* values from 2 to 3, and small *J* values from 3 to 4, was similar as for residue **A** (Figure 2). However, the ^13^C chemical shift for C-2 was found at 52.51 ppm, in the region for amino sugars. Proven by a 2D HMBC cross-peak from H-2 to a carbon in the carboxylic region, the amino function was acetylated and **D** is therefore α-Gal*p*NAc (Figure 3A). The anomeric proton of **E** was close to the residual solvent signal (Figures S4 and S5). The spin system showed the same features, like sugars **D** or **A**, so **E** has a *galacto*-configuration. A 2D HMBC cross-peak at the carbon shift from C-4 identifies H-5, which, in the 2D DQF-COSY, showed a correlation in the aliphatic region to a doublet at 1.15 ppm. The spin simulation manifested all features of the 6-deoxy sugar **E** as α-Fuc*p* (Figure 2). Going to higher field, the next signal was completely covered by the residual solvent (Figures S4 and S5). A 2D HMBC cross-peak from the anomeric proton to a CH signal at a carbon shift of 55.7 ppm identified **F** as a 2-amino-2-deoxy sugar, and the corresponding proton at this carbon shift was located at 4.74 ppm, covered by the solvent as well. This proton and also another doublet at 3.74 ppm had long-range correlations to carbonyl carbon signals in the HMBC spectrum. Monosaccharide **F**, thus, has features of 2-*N*-acetylamino-2-deoxy-uronic acid (Man*p*ANAc). The analysis of the coupling network in the 1D TOCSY spectrum together with the spin simulation finally resulted in Man*p*ANAc in β-configuration, due to a CH coupling constant of 161.5 Hz (Figure 2). At the right side of the residual solvent, the anomeric proton **G** was clearly visible, with the *J* coupling in the range of the line width and, thus, not resolved. As starting point for the selective 1D TOCSY, the complete spin system can be derived by applying a long spin-lock period. The analysis together with the spin simulation identified sugar **G** as Man, again in the β-configuration derived from a heteronuclear coupling constant of 160.4 Hz (Figure 2). Finally, starting from the anomeric proton of building block **H** with the large splitting of 8 Hz, the pathway to H-2 and further to H-3 can be easily followed in the 2D DQF-COSY experiment, and the fine structure of these cross-peaks displayed only large *J* values, implicating a glucose-type sugar (Figure 2). This was confirmed with the selective 1D TOCSY spectrum and, as a result, residue **H** is β-Glc*p*. All identified aldohexoses formed pyranoses, evidenced by appropriate 2D HMBC cross-peaks from the anomeric proton to C-5 or from the anomeric carbon to H-5, respectively.

The inter-glycosidic linkage information was derived from appropriate cross-peaks in a 2D HMBC experiment (Figure 3A), assisted by a 2D NOESY spectrum (Figure 3B). Further confirmation was obtained from the ^13^C chemical shift, as the involved carbons should exhibit a down-field shift up to 10 ppm due to glycosylation (Table 2). Residue **A** had an HMBC cross-peak from H-1 to C-3 of building block **F** (Figure 3A). All carbons except C-1 had chemical shifts in the range of unsubstituted carbons.

In total, these data provided the following information. Sugar **A** was the terminal residue at the non-reducing site. The oligosaccharide started from this end with the disaccharide unit α-Gal*p*-(1→3)-β-Man*p*ANAc. The carbon chemical shift of C-3 from unit **F**, which was deshielded due to glycosylation with **A**, was similar as for C-4, suggesting another substitution site (Table 2). Since an HMBC cross-peak could be found from this C-4 to the anomeric proton from sugar **G** (Figure 3A), β-Man*p*ANAc **F** was a branching point of the glycan. Like **A**, this β-Man*p* **G** was the end of this side chain, since none of the carbons except C-1 were shifted due to the absence of glycosylation (Table 2). The next residue in the direction of the reducing end was identified by the HMBC cross-peak from H-1 of sugar **F** to C-4 of the Fuc*p* **E**. Unit **E** was disubstituted, shown by the HMBC cross-peak from C-3 to the anomeric proton of the α-Gal*p*NAc **D**. Next, H-1 from **E** had a long-range correlation to C-4 of the Glc*p* **H**. Finally, an HMBC cross-peak linked H-1 of **H** with C-4 of the GlcA **B** (Figure 3A). The next connectivity going onward from **B** was not to an aldose, although one residue would be still available, namely the α-(2-O-Me)-Rha*p* **C**. On the other hand, from both of these anomeric protons from **B** and **C**, HMBC cross-peaks to two individual carbons were visible at around 82 ppm (Figure 3A), of which none corresponded to the sugars analyzed so far. As the glycan was obtained by β-elimination under reductive conditions, the last part of the oligosaccharide was an alditol denoted as **I**, glycosylated with **B** at position 2 and with **C** at position 4 (Table 2). The nature of the starting hexose leading to this sugar alcohol could not be deduced from the NMR data at this point. As a cross check for all inter-glycosidic HMBC cross-peaks, a 2D-NOESY spectrum affirmed all connectivities with cross-peaks between the anomeric protons and the appropriate protons at the linkage position (Figure 3B).

The combined data of the NMR analysis (Table 2) and simulated ^1^H NMR spectra of the sugar units (Figure 2) reveal that the isolated glycan from *B. fragilis* is a nonasaccharide (Figure S6), which agrees with the experimental mass of 1571.8 Da. While the biochemical data are in full support of the presence of l-fucose, the absolute configuration of the rhamnose unit remains to be firmly established and has only been tentatively assigned to l-configuration in Figure S6.

### 3.3. Determination of the Reducing-End Sugar of the Bacteroides fragilis O-Glycan

**Figure 4:**
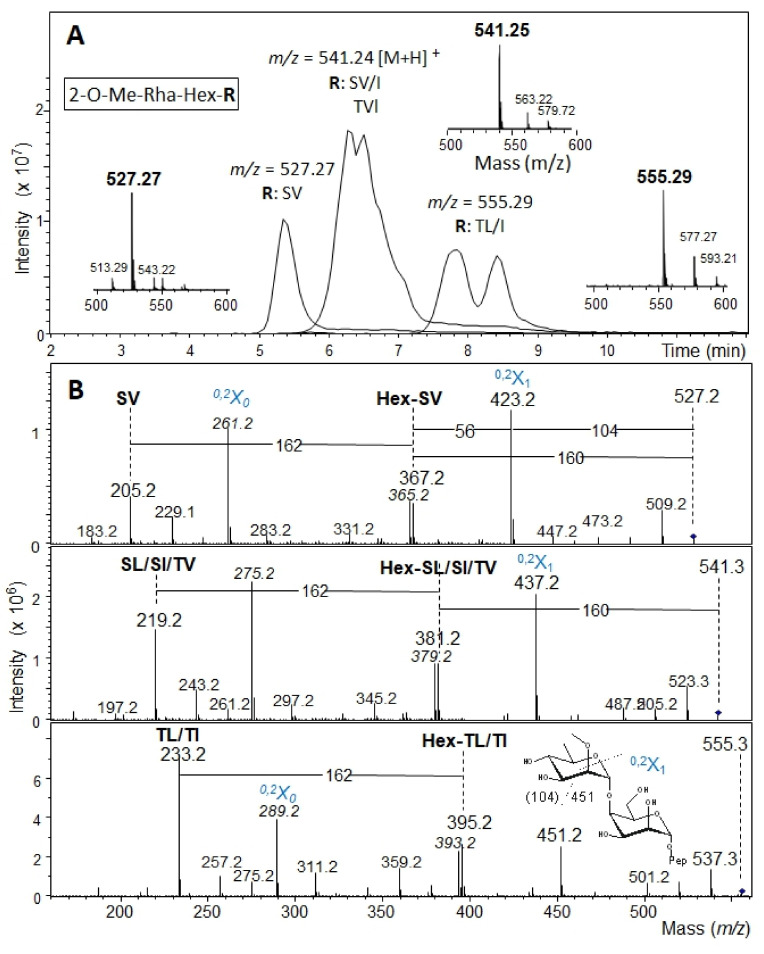
(**A**) Overlaid extracted ion chromatograms of PGC-LC-ESI-MS analysis of *B. fragilis* Δ*BF4306* glycopeptides obtained by PCG-HPLC fractionation. A mass of 527.27 Da indicates glycopeptide 2-O-Me-Rha-Hex-Ser-Val (fraction f28), of 541.24 Da glycopeptide 2-O-Me-Rha-Hex-Ser-Leu/Ile or 2-O-Me-Rha-Hex-Thr-Val (fraction f32), and of 555.29 Da glycopeptide 2-O-Me-Rha-Hex-The-Leu/Ile (fraction f36). Small insets show the respective MS^1^ spectrum. (**B**) The corresponding MS^2^ spectra confirm the glycopeptide nature of *B. fragilis* Δ*BF4306* glycopeptides f28, f32, and f36 as obtained by PGC-HPLC separation and fractionation in the previous purification. The spectra exhibit prominent peaks from cross-ring cleavages. Peaks explicable by rearrangements—as frequently observed in positive mode fragment spectra—are labeled with italic numbers. Cross-ring fragments indicated in blue are described as proposed by Domon and Costello [47].

To identify the reducing-end sugar of the *B. fragilis* nonasaccharide, glycopeptides were prepared from the *B. fragilis* Δ*BF4306* mutant, which produces a truncated *O*-glycan containing only two of the nine protein-linked sugar residues [7]. Glycopeptides, pre-purified by size exclusion chromatography, were further separated via PGC-HPLC and collected fractions screened by PGC-LC-MS. Three dominant fractions identified by MS and MS^2^ (Figure 4) were chosen for subsequent NMR analysis. In the MS-spectra, peaks with *m*/*z* = 527.27 [M + H]^+^ conforming to 2-O-Me-Rha-Hex-Ser-Val (fraction f28), *m*/*z* = 541.24 [M + H]^+^ conforming to 2-O-Me-Rha-Hex-Ser-Leu/Ile or 2-O-Me-Rha-Hex-Thr-Val (fraction f32), and *m*/*z* = 555.29 [M + H]^+^ conforming to 2-O-Me-Rha-Hex-Thr-Leu/Ile (fraction f36) were observed (Figure 4A). CID MS^2^ in the ion trap typically left the dipeptide portion intact with additional fragments showing mass increments of a hexose plus a methylated deoxyhexose (Figure 4B). Thus, the three glycopeptide samples (f28, f32, f36) differed in the peptide portion while containing the identical disaccharide, as expected. The MS^2^ spectra exhibited signs of considerable rearrangements as usual for positive mode CID [44,45,46].

For f28, f32, and f36, ^1^H NMR revealed a high peptide background with high coverage of the carbohydrate chemical shift region; furthermore, the amount of the samples was very low (Figure S7). In the anomeric region of the ^1^H NMR spectrum, the signal with the highest intensity was at approximately 5.00 ppm in all three spectra. Additional signals are visible in upfield direction, but they differed in number, intensity, and chemical shift in the three preparations. The main reason for this heterogeneity seems to originate from the different peptides. Omitting the signals near baseline in the spectra, f28 showed only one additional anomeric proton at approximately 4.88 ppm (Figure S7, upper trace). In f32, three anomeric signals were visible for the second sugar, one identical to that in f28, suggesting that the disaccharide-peptide structure from f28 is also present in f32. The two remaining anomeric protons derived either from the sugar linked to the Ser-Leu or the Ser-Ile amino acid sequence (Figure S7, middle trace). Finally, the preparation with the lowest intensity, f36, showed two anomeric protons for the second sugar, which can be attributed to two different peptides (Figure S7, lower trace). As f28 was the most promising candidate with regard to intensity and homogeneity in the peptide portion, it was chosen for detailed NMR analysis.

Starting with the anomeric proton of the first sugar at 5.00 ppm, one dominant TOCSY cross-peak under standard spin-lock conditions of 100 ms occurred at 3.61 ppm. Analysis of the appropriate frequency in the 1D ^1^H NMR spectrum revealed the corresponding multiplet was formed only by small *J* couplings, suggesting a mannose-type sugar. The 2D TOCSY trace at this chemical shift showed the rest of the spin system, with one signal located in the aliphatic region and a CH_3_ group as a doublet at 1.24 ppm. Applying a selective 1D TOCSY experiment by excitation of the methyl protons followed by a spin simulation, this sugar was identified as a Rha*p*. This Rha*p* is substituted as the carbon at position 2 was deshielded to 83.13 ppm, and an HMBC cross-peak was present at this ^13^C frequency connecting a sharp single line proton signal at 3.45 ppm. The anomeric configuration was α as proven by a heteronuclear *J* coupling of 169.7 Hz. Taken together, these data showed that this building block is an α-(2-O-Me)-Rha*p* and therefore matches unit **C** of the intact *B. fragilis* glycan. For this reason, in glycopeptide fraction f28, this residue was named **C’** (Figure S8). The 2D TOCSY trace starting from the anomeric proton of the second sugar at 4.88 ppm was similar as for the first sugar. Again, essentially only one cross-peak was visible at a chemical shift of 3.9 ppm. The coupling pattern of this H-2 originated only from small *J* values, implicating a mannose-type sugar. Access to the complete spin system for this unit was only possible by a selective 1D TOCSY experiment starting from the anomeric proton using a long 300 ms spin-lock. Based on the interpretation of the 1D TOCSY spectrum, a spin simulation confirmed the Man*p* (Figure S8). The anomeric configuration was α according to a heteronuclear *J* coupling of 170.3 Hz. This α-Man*p* residue represents the reducing-end sugar before the reductive β-elimination of the *B. fragilis* nonasaccharide and was named **I’**.

**Figure 5:**
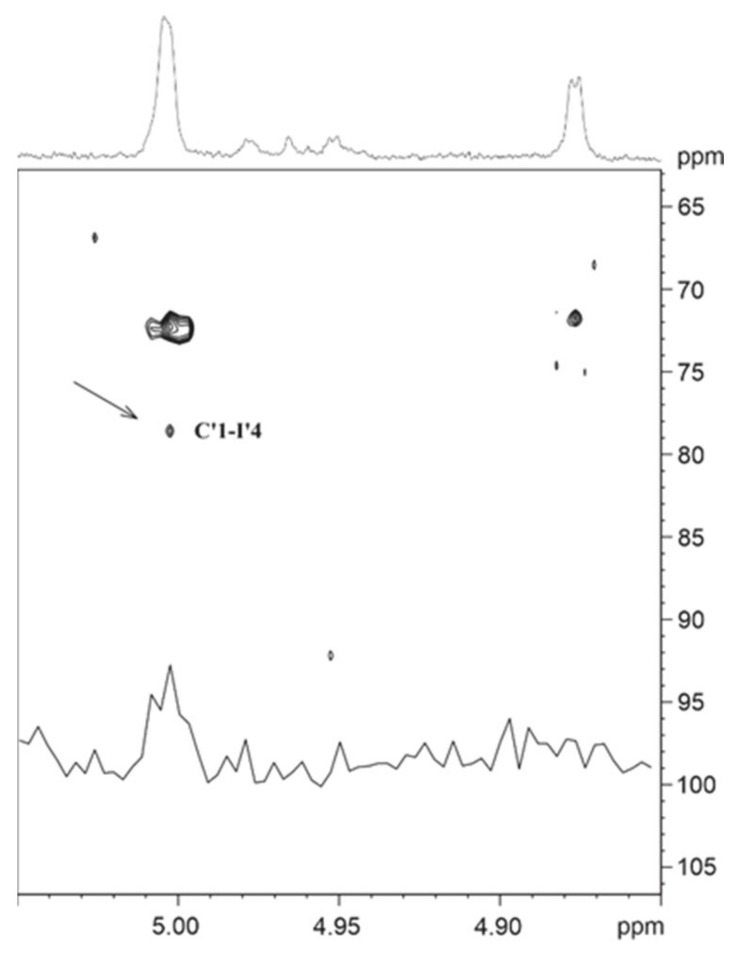
Region of the 2D-HMBC spectrum from the *B. fragilis* Δ*BF4306* glycopeptide f28. The arrow marks the inter-glycosidic cross-peak from the anomeric proton of Rha **C’** to carbon 4 of the Man **I’**. The inserted trace shows the ^1^H row at the carbon frequency of the inter-glycosidic cross-peak **C’**1-**I’**4.

Due to the low sample amount and large peptide background signals in the spectra, the elucidation of the linkage between the two monosaccharides of the disaccharide was challenging. In the 2D NOESY spectrum, cross-peaks could be traced from H-1 of the Rha*p* to signals at 3.77 and 3.90 ppm. The positions of the first chemical shift H-3 of Rha*p* and of H-4 of Man*p* were located more or less on top of each other. As the anomeric configuration of the Rha is α, a cross-peak from the equatorial H-1 to the axial H-3 is rather unlikely. In a 2D ROESY experiment with 200 ms mixing time, this cross-peak was more intense, which enabled the analysis of the cross-peak fine structure. The shape implied a triplet structure, thus resulting from two *J* couplings of similar size, which only fits H-4 of the Man*p*. The second cross-peak at 3.90 ppm with a much lower intensity was H-3 of the Man*p*. These results revealed either a 3 or a 4 linkage from Rha*p* to the Man*p*, although the higher cross-peak intensity representing a shorter distance between the two involved protons at the linkage site suggested a higher probability for position 4. In addition, the linkage from the α-(2-O-Me)-Rha*p* **C** to the alditol in the previously analyzed oligosaccharide is also position 4 (compared with Figure S6). Final verification was possible with an HMBC spectrum showing a cross-peak from the anomeric proton of the Rha*p* to C-4 of the Man*p* (Figure 5). These data prove that the missing hexose, which was reduced to the sugar alcohol during preparation of the *B. fragilis* glycan, is α-Man*p*.

**Figure 6:**
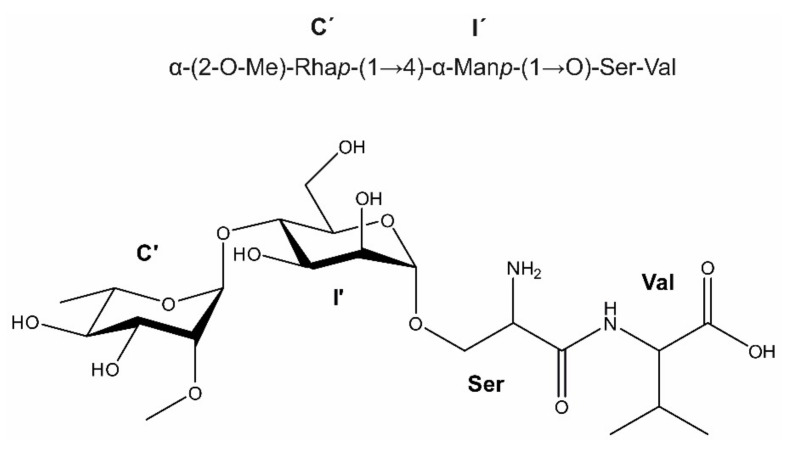
Structure of the *B. fragilis* Δ*BF4306* glycopeptide f28, revealing the glycan-peptide linkage. The absolute configuration of the rhamnose unit **C‘** remains to be firmly established and has only been tentatively assigned to l-configuration.

As shown by the mass spectrometry analysis, the disaccharide portion of f28 had an *O*-glycosidic linkage to a dipeptide fragment. Starting from the sugar portion, the corresponding peptide-Man linkage was revealed in the 2D NOESY spectrum, showing a cross-peak from H-1 of the α-Man*p* **I’** to a chemical shift of 3.93 ppm, one of the protons from the CH_2_ group of serine. From this chemical shift, the rest of this amino acid spin system could be assigned. For the valine, two doublets at 0.95 and 0.91 could be assigned as methyl groups, and the rest of the amino acid spin system is accessible in the 2D TOCSY spectrum. The simulated ^1^H NMR spectra of the two sugar units from the glycopeptide f28 is shown in Figure S8, and the elucidated structure of the glycopeptide is shown in Figure 6.

**Figure 7:**
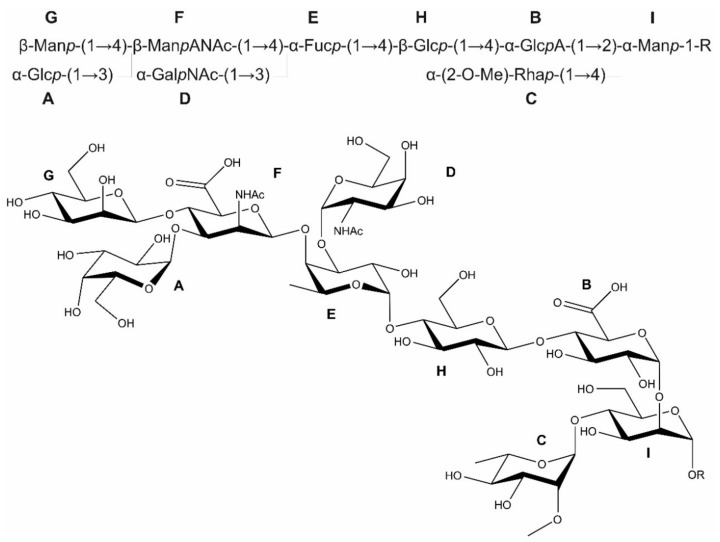
Complete structure of the *B. fragilis* *O*-glycan by combining the 700 MHz NMR spectroscopy results of the structure elucidation from *B. fragilis* wild-type *O*-glycan released from glycopeptides by reductive β-elimination and from the *B. fragilis* Δ*BF4306* glycopeptide f28. R at the reducing end denotes the peptide portion. While the biochemical data are in full support of the presence of l-fucose, the absolute configuration of the rhamnose unit **C** remains to be firmly established and has only been tentatively assigned to the l-configuration. **A**–**I** refers to the individual sugars as described in the text.

The complete structure of the *B. fragilis* *O*-glycan including the linkage sugar to the protein portion is shown in Figure 7. All NMR data of the nonasaccharide preparations from *B. fragilis* wild-type and the disaccharide glycopeptide f28 from a *B. fragilis* Δ*BF4306* mutant are summarized in Table 2.

### 3.4. Bacteroidetes FucTs Can Cross-Complement

**Figure 8:**
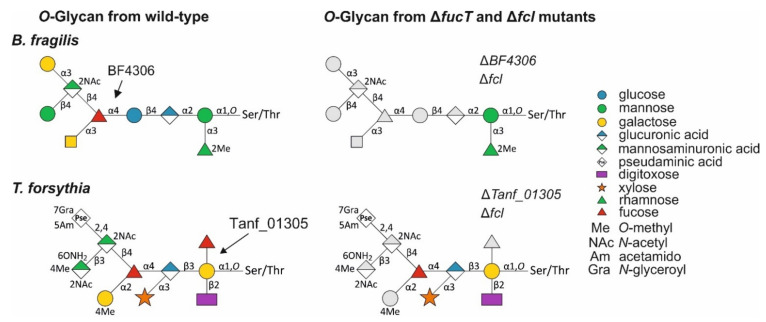
Comparison of the *O*-linked glycan structures from *B. fragilis* wild-type (this study), *B. fragilis* Δ*BF4306* (this study)*, B. fragilis* Δ*gmd-fcl*Δ*fkp* [16], *T. forsythia* wild-type [10], *T. forsythia* Δ*Tanf_01305* [10], and *T. forsythia* Δ*fcl* (this study). The severe truncation of the glycans upon deletion of the *fucT* or the *fcl* gene is indicated in grey. Glycan structures are drawn according to the symbol nomenclature of glycans SNFG [48].

The structures of the *T. forsythia* and *B. fragilis* *O*-glycans suggest that their FucTs transfer Fuc to different hexose sugars (Figure 8). Here, we tested the ability of each of these FucTs to complement mutants in the heterologous species. In addition, we tested the ability of the FucT from *P. heparinus*, Phep_4048, of a phylogenetically distant Bacteroidetes species whose FucT is only 48.1% and 48.6% similar to the FucT of *B. fragilis* and *T. forsythia* respectively, to complement these mutants. As read-out for glycosylation, we used specific antisera to the protein portion of the abundant *T. forsythia* S-layer glycoproteins TfsA and TfsB [8] and the protein portion of the *B. fragilis* glycoprotein BF2494, an abundant soluble periplasmic protein [37].

**Figure 9:**
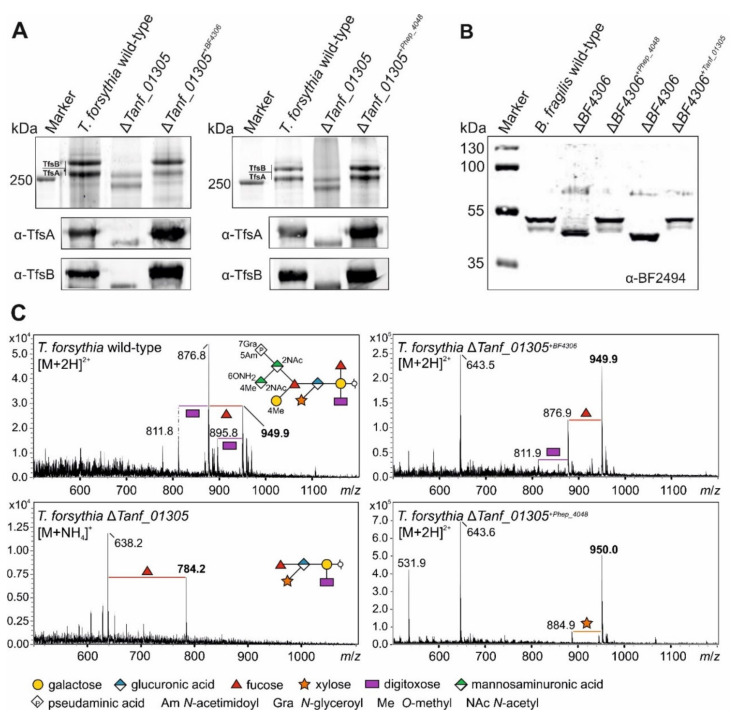
(**A**) Analysis of crude cell extracts from *T. forsythia* ATCC 43037 wild-type, the FucT-deficient mutant (Δ*Tanf_01305*) and cross-complemented strains (*ΔTanf_01305*^+*BF4306*^; *ΔTanf_01305*^+*Phep_4048*^) after separation on 7.5% SDS-PAGE gels. Top panels—CBB staining of the S-layer glycoproteins (labeled TfsA and TfsB) showing the downshifts resulting from glycan truncation in the mutant. S-layer glycoprotein bands were further processed for MS analyses. Bottom panels—same samples processed for Western immunoblot analysis probed with a α-TfsA and α-TfsB antiserum, respectively. PageRuler Plus Prestained Protein Ladder (Thermo Fisher Scientific) was used as a protein molecular weight marker. (**B**) Western immunoblot analyses of crude cell extracts from *B. fragilis* wild-type, the FucT-deficient mutant (Δ*BF4306*) and cross-complemented strains (Δ*BF4306*^+*Phep_4048*^; Δ*BF4306*^+*Tanf_01305*^) probed with α-BF2494 antiserum. (**C**) ESI-MS sum spectra of β-eliminated TfsB *O*-glycans from *T. forsythia* wild-type, Δ*Tanf_01305* and cross-complemented strains. The glycan structures of the signals corresponding to the largest mass (bold *m*/*z* values) are drawn according to the symbol nomenclature of glycans SNFG [48]. *O*-glycan signals detected for the mutant and cross-complemented strains were assigned based on the *m*/*z* mass differences corresponding to the loss of individual sugar units and/or modifications. Peak intensities are shown on the *y* axis.

Western immunoblots using α-TfsA and α-TfsB antibodies (Figure 9A) and α-BF2494 antiserum (Figure 9B) showed the reduction in the size of the glycoproteins in the Δ*fucT* mutants—indicative of glycan truncation in these bacteria. The *T. forsythia* glycan was reduced from a decasaccharide to a pentasaccharide [9] and the *B. fragilis* nonasaccharide was reduced to a disaccharide [7] (Figure 8). When the heterologous genes were added to these mutants in trans, the glycoproteins migrated identical to those in the wild-type bacteria in SDS-PAGE. For *T. forsythia*, the detection of the MW-shifts was also possible using CBB-staining due to the cellular abundance of the S-layer glycoproteins and their large size (Figure 9A). In addition, MS analysis of released S-layer *O*-glycans verified that cross-complementation of *T. forsythia* Δ*Tanf_01305* with BF4306 and Phep_4048, respectively, restored the complete *T. forsythia* wild-type *O*-glycan (Figure 9C).

### 3.5. A T. forsythia GDP-l-Fucose Synthase fcl Knock-Out Strain Produces the Same Truncated O-Glycan Phenotype as the FucT-Deficient Strain

**Figure 10:**
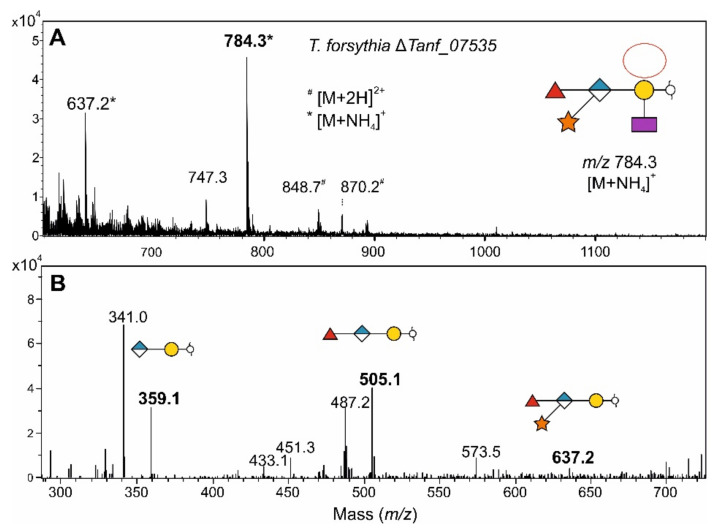
MS analysis of the β-eliminated TfsA *O*-glycans from *T. forsythia* Δ*Tanf_07535* (Δ*fcl*). In the deconvoluted ESI-MS sum spectrum (**A**) of β-eliminated TfsA *O*-glycans from *T. forsythia* Δ*Tanf_07535* (Δ*fcl*), the glycan structure of the signal corresponding to the *m*/*z* 784.3 value is drawn according to the symbol nomenclature of glycans SNFG [48]. *, [M + NH_4_]^+^; ^#^, [M + 2H]^2+^. The MS^2^ spectrum (**B**) confirms the glycan nature of the *m*/*z* 784.3 peak [10]. Peak intensities are provided on the *y* axis.

The *T. forsythia* *O*-glycan contains two Fuc residues, of which only the galactose-bound, inner Fuc is affected in the Δ*Tanf_01305* mutant (Figure 8), as previously demonstrated by detailed MS^2^ analyses of that mutant [9]. Based on the loss of fucosylation in a *B. fragilis* mutant deficient in GDP-l-Fuc biosynthesis [15] and the demonstrated functional homology of the FucTs from *B. fragilis* and *T. forsythia*, we reasoned that the Fuc residue branching from the protein-linked Gal of the *T. forsythia* glycan is l-Fuc, and the GlcA-linked Fuc at the branching point of the glycan is d-Fuc. To confirm this, we created a *T. forsythia* Δ*Tanf_07535* mutant defective in the GDP-l-Fuc synthase Fcl, which catalyzes the conversion of GDP-d-Man to GDP-l-Fuc [18]. Unlike *Bacteroides* and *Parabacteroides* species, where there are both *de novo* and salvage pathways for the synthesis of GDP-l-Fuc [16], *T. forsythia* does not contain the gene encoding Fkp of the salvage pathway, and therefore, deletion of *fcl* is sufficient to abrogate GDP-l-Fuc synthesis and prevent incorporation of l-Fuc into oligo- and polysaccharides. As expected, the ESI-MS spectrum of the *O*-glycan of *T. forsythia* Δ*fcl* showed the same phenotype as *T. forsythia* Δ*Tanf_01305*, revealing the significant *m*/*z* = 784.3 [M + NH_4_]^+^ indicative of a pentasaccharide lacking the Fuc branching from the protein-linked Gal (Figure 10). These data showed that Tanf_01305 transfers an l-Fuc and that the Fuc attached to the GlcA at the branching point of the *T. forsythia* glycan is likely a d-Fuc.

### 3.6. A T. forsythia GDP-l-Fucose Synthase fcl Knock-Out Strain Produces the Same O-Glycan Phenotype as the FucT-Deficient Strain

Although all three FucT orthologs can cross-complement, we showed that two of these FucTs create different linkages, one to Gal (*T. forsythia*) and one to Glc (*B. fragilis*) (Figure 8). To further demonstrate that Tanf_01305, BF4306, and Phep_4048 are l-FucTs, and to determine if they are specific for Fuc transfer to hexose, an in vitro FucT assay was performed. Each protein was translationally fused to MBP and produced in and purified from *E. coli*. SDS-PAGE analysis of these purified recombinant proteins showed that rPhep_4048 and rBF4306 have lower MW forms in addition to the full-length form, likely due to degradation (Figure S9).

**Figure 11:**
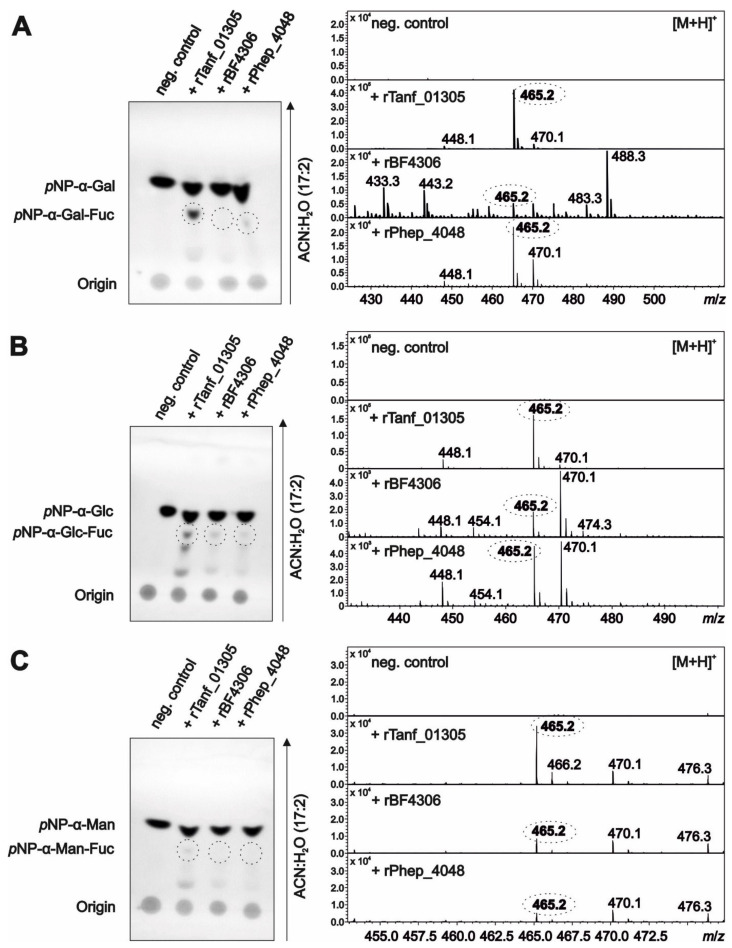
In vitro fucosyltransferase activity assays on *p*NP-α-l-hexoses. Developed TLC plates in combination with MS analysis show the formation of (**A**) *p*NP-α-d-Gal-Fuc, (**B**) *p*NP-α-d-Glc-Fuc, and (**C**) *p*NP-α-d-Man-Fuc when using recombinant FucTs from *T. forsythia* (rTanf_01305), *B. fragilis* (rBF4306), and *P. heparinus* (rPhep_4048) respectively. To determine the product masses corresponding to the detected spots on TLC plates, the reaction product (marked by a dashed circle) was eluted from the TLC plate and analyzed by ESI-MS. The mass corresponding to a *p*NP-α-d-Hex-Fuc product (465.2 *m*/*z*) is written in bold in a dashed circle. Peak intensities are given on the *y* axis. Negative controls were assay mixtures without enzyme.

All enzymes were tested for their ability to fucosylate different *p*NP-hexose acceptor substrates, including *p*NP-α-Gal, *p*NP-α-Glc and *p*NP-α-Man and, in addition, *p*NP-α-GlcA (GlcA is the attachment site of the second Fuc in the *T. forsythia* *O*-glycan structure; Figure 8), *p*NP-β-Xyl and *p*NP-β-GlcNAc (negative controls), with GDP-l-Fuc serving as the Fuc donor. All three enzymes transferred l-Fuc to *p*NP-α-Gal (Figure 11A), *p*NP-α-Glc (Figure 11B), and *p*NP-α-Man (Figure 11C), albeit with different preferences according to semiquantitative detection of the reaction products on TLC plates and by ESI-MS, evidenced by *m*/*z* of *p*NP-Hex-Fuc = 465.2 [M + H]^+^. Low activities could be due to the recombinant form of the proteins and/or suboptimal reaction conditions. rTanf_01305 transferred an l-Fuc residue to all three tested acceptor substrates with *p*NP-α-Gal as the best substrate—confirming reactivity on its native acceptor in the *T. forsythia* *O*-glycan—and *p*NP-α-Man as the least favorable substrate (*p*NP-α-Gal > *p*NP-α-Glc > *p*NP-α-Man). rBF4306 demonstrated very low activity with all substrates; *p*NP-α-Glc was the best substrate, which is in agreement with the finding that l-Fuc is linked to a Glc residue in the *B. fragilis* *O*-glycan structure (Figure 8). rBF4306 also demonstrated slight transfer to *p*NP-α-Man substrate, while only traces of modified *p*NP-α-Gal were detected. However, cross-complementation showed that in the native organism, BF4306 complements the function of Tanf_01305. rPhep_4048 showed comparable preferences for the *p*NP-hexose acceptor substrates as rTanf_01305, but was only minimally active. The structure of the *P. heparinus* glycan has not been determined and therefore its natural preferred substrate is unknown; however, these data combined with the cross-complementation data suggest that rPhep_4048 links l-Fuc to a hexose residue of its *O*-glycan. Neither *p*NP-α-GlcA, *p*NP-β-Xyl, nor *p*NP-α-GlcNAc was a suitable acceptor substrate for any of the three enzymes (Figure S10).

### 3.7. Fucosidase Treatment of pNP-α-d-Gal-Fuc Suggests an α1,6-Linkage of the l-Fuc in the T. forsythia O-Glycan

Since the linkage between the l-Fuc residue and the reducing-end Gal in the *T. forsythia* *O*-glycan structure is unknown, we used the *p*NP-α-Gal-Fuc reaction product from the rTanf_01305 activity assay to elucidate this linkage (Figure 11A). We purified the compound from the in vitro reaction by preparative TLC and used various fucosidases of known specificities to investigate the linkage. We used commercially available fucosidases (α1,2 fucosidase, α1,3/4 fucosidase, and α1,2/4/6 fucosidase O) as well as *T.* *forsythia* fucosidase TfFuc1 that was recently characterized in our laboratory to be an α1,2-fucosidase with additional α1,6 specificity on small unbranched substrates [35]. TLC showed that treatment with α1,2/4/6 fucosidase O and rTfFuc1 resulted in cleavage of the terminal Fuc from *p*NP-α-d-Gal-Fuc (Figure S11). From these data, together with the knowledge of the *T. forsythia* *O*-glycan structure where the protein-linked Gal residue is substituted at the C2 with a Dig residue, we conclude that the linkage between l-Fuc and the Gal residue is most likely α1,6.

## 4. Discussion

Members of the phylum Bacteroidetes are colonizers of numerous habitats on Earth [4]. They are among the major members of the microbiota of animals, especially in the gastrointestinal tract, can act as pathogens, and are frequently found in soils, oceans, and freshwater. Despite these diverse ecological niches, we revealed commonalities in Bacteroidetes with regards to the biosynthesis of *O*-linked glycoproteins involving a conserved FucT that is widely distributed in the phylum (Figure 1).

For *B. fragilis* and *T. forsythia*, the presence of a general protein *O*-glycosylation system targeting various cellular proteins was demonstrated previously and showed the biological importance of protein glycosylation [7,22,49]. In these bacteria, the *O*-glycan is a complex oligosaccharide with both a core (inner glycan) and outer glycan. In *Bacteroides* species, the outer glycan enzymes are encoded by the *lfg* region [7,10], whereas the genes involved in core glycan synthesis are located elsewhere on the bacterial genome.

To determine the role of the conserved FucT from *T. forsythia* (Tanf_01305), *B. fragilis* (BF4306), and *P. heparinus* (Phep_4048) as representatives of the phylum, we first elucidated the complete structure of the *B. fragilis* *O*-glycan by 1D and 2D NMR spectroscopy and revealed that it is a branched nonasaccharide with a Fuc residue serving as a branching point in is backbone structure (Figure 7). The *O*-glycan structure analysis of the *B. fragilis* Δ*BF4306* mutant, in contrast, revealed a heavily truncated structure devoid of Fuc (Figure 6 and Figure 10) further supporting that BF4306 is a FucT. These data also support the previous finding that the core glycan is devoid of Fuc and that the BF4306 enzyme is essential for outer glycan biosynthesis. Interestingly, the branched disaccharide α-2-O-Me-Rha*p*-(1→3)-Man*p*-(1→*O* of the *B. fragilis* core glycan (this study) is identical with those of the *Elizabethkingia meningoseptica* hydrolase glycoproteins, according to MS-based evidence [50], and *P. heparinus* heparinase I, however without provision of a detailed structural analysis [51].

The observation that deletion mutants of conserved FucT in *B. fragilis* Δ*BF4306* and *T. forsythia* Δ*Tanf_01305* leads to glycans that are lacking not only the Fuc residue transferred by the FucT but also proximal sugar residues in the *O*-glycans—i.e., to a disaccharide in *B. fragilis* and a pentasaccharide in *T. forsythia* [7,10] (compared with Figure 10)—raises questions about the biosynthetic pathway for the protein-linked *O*-glycans. For instance, it might involve a signalling function of the FucT to upstream-acting biosynthetic enzymes or the FucT might be part of a multienzyme complex. To investigate the latter possibility, pull-down experiments with the different FucTs are currently underway in our laboratory.

The analyses of the Δ*fcl* mutant of *T. forsythia* revealed that this *O*-glycan likely contains d-Fuc in addition to l-Fuc. In most cases, FucTs catalyze an inverting reaction in which GDP-β-l-Fuc serves as a donor substrate; this is de novo synthesized from GDP-d-Man involving the enzymes Gmd (GDP-mannose-4,6-dehydratase) and Fcl (GDP-keto-6-deoxymannaose 3,5-epimerase/4-reductase) [18]. Rare examples of bacterial carbohydrate structures contain d-configurated Fuc—e.g., the O-specific polysaccharide of the LPS from *Mesorhizobium huakuii* strain S-52 [52], the O-antigen of *Aggregatibacter actinomycetemcomitans* (previously, *Actinobacillus actinomycetemcomitans*) Y4 (serotype b) [53] or the S-layer glycan from *Geobacillus tepidamans* GS5-97^T^ [54]. For the biosynthesis of these, TDP-d-Fuc serves as the d-Fuc donor and is produced along the RmlA (glucose-1-phosphate thymidylyltransferase)/RmlB (dTDP-glucose-4,6-dehydratase)/Fcd (dTDP-4-dehydro-6-deoxyglucose reductase) pathway [53,54]. A Δ*fcl* mutant of *T. forsythia* (Figure 8) and of *B. fragilis* [16] (Figure 8) clearly revealed that GDP-l-Fuc is the substrate for the conserved FucTs, allowing the assignment of the targeted Fuc residue in the *O*-glycans as an l-Fuc. As the second Fuc present in the *T. forsythia* glycan (Figure 10) was not affected by the lack of GDP-l-fucose in the *T. forsythia* Δ*fcl* mutant, it is likely transferred by another FucT requiring TDP-d-Fuc. This would imply that the *T. forsythia* *O*-glycan is among the rare examples of bacterial carbohydrate structures that contain both an l-Fuc and a d-Fuc residue.

The conserved FucTs Tanf_01305, BF4306, and Phep_4048 are functional orthologs based on the successful cross-complementation of the *T. forsythia* Δ*Tanf_01305* and the *B. fragilis* Δ*BF4306* mutants with the non-native *fucT* genes leading to the restoration of the respective wild-type glycans (Figure 9).

We showed in an in vitro enzyme assay that BF4306, Tanf_01305, and Phep_4048 are active on various *p*NP-hexose acceptor substrates, including *p*NP-Gal, *p*NP-Glc, and *p*NP-Man (Figure 11). Under the experimental conditions used, the enzymes had different preferences for the *p*NP-hexose substrates, indicative of substrate promiscuity of the conserved Bacteroidetes FucTs. Further, these FucTs might also exhibit some degree of promiscuity with regards to the linkage specificity. BF4306 is an α1,4-FucT as concluded from the linkage data of the Fuc residues in the *B. fragilis* *O*-glycan (Figure 7). The linkage information of the l-Fuc in the *O*-glycan structure of *T. forsythia* is missing, however, the data of this study suggest that Tanf_01305 is an α1,6 FucT (Figure S11). Thus, the conserved Bacteroidetes FucTs investigated within the frame of this study might represent the first bacterial FucTs with α1,4/6 specificity. The α1,3/α1,4 FucTs, which are mostly classified as GT10 family enzymes and have a DXD motif, are evolutionary distinct form the superfamily of α1,2/α1,6/O-FucTs. The Bacteroidetes FucT family described here has a DXD motif, fitting with α1,4-linked Fuc observed in the *B. fragilis* *O*-glycan, but not with α1,6-linked Fuc deduced for the *T. forsythia* *O*-glycan. Furthermore, these new FucTs group as GT2 and not with other known FucTs CAZy families.

These remaining questions illustrate the need for further research on FucTs to obtain a more detailed understanding of their functional capabilities. Furthermore, FucTs are promising tools for biosynthetic glycobiology. Significant examples of Fuc-containing glycoconjugates include the biotechnological production of fucosylated human milk oligosaccharides [55], engineering of cancer vaccines presenting Fuc-containing tumor-associated carbohydrate antigens (such as Lewis antigens, Globo H, fucosyl-GM1) [56], and chemoenzymatic synthesis of cholera toxin inhibitors [57]. Therefore, a more detailed analysis of the activity of the FucTs is warranted to understand their true potential in glycoengineering.

## 5. Conclusions

1. The structure of the protein-linked *O*-glycan of *B. fragilis* NCTC 9343 was elucidated by 1D and 2D NMR spectroscopy and shown to be a complex, branched nonasaccharide with an l-Fuc residue in the backbone structure.
2. The described l-FucT is relatively conserved among different members of the bacterial phylum Bacteroidetes. Functional orthologs of the FucT BF4306 were demonstrated in the periodontal pathogen *Tannerella forsythia* (Tanf_01305) and the soil bacterium *Pedobacter heparinus* (Phep_4048), using in vivo cross-complementation and an in vitro enzyme assay.
3. Enzymatic analyses revealed that these l-FucTs exhibit relaxed acceptor substrate specificity transferring l-Fuc from GDP-l-Fuc to galactose, glucose, and mannose residues, with α1,4/6 linkage specificity.
4. Given the biological importance of fucosylated carbohydrates, the Bacteroidetes l-FucTs are promising candidates for glycobiology applications.

## Figures and Tables

**Table 1:** Bacterial strains and plasmids used in this study.

Strain or Plasmid	Genotype and Use or Description	Source or Reference
***Escherichia coli* Strains**		
DH5α	F^−^ Φ80*lac*Z∆M15 ∆(*lac*ZYA-*arg*F) U169 *rec*A1 *end*A1 *hsd*R17 (rK–, mK+) *pho*A *sup*E44 λ- *thi*-1 *gyr*A96 *rel*A1; cloning strain	Thermo Fisher Scientific
BL21(DE3)	F*^−^ ompT hsdS_B_* (*r_B_^−^m_B_^−^*) *gal dcm* (DE3); expression strain	Thermo Fisher Scientific
***Tannerella forsythia* Strains**		
ATCC 43037	Type strain, wild-type	ATCC; [26]
ATCC 43037 Δ*Tanf_01305*	Δ*Tanf_01305*:(P*erm*)*-ermF;* knock-out strain of *Tanf_01305*	[10]
ATCC 43037 Δ*Tanf_01305^+BF4306^*	Δ*Tanf_01305*::*BF4306 cat*; cross-complemented knock-out strain	This study
ATCC 43037 Δ*Tanf_01305^+Phep_4048^*	Δ*Tanf_01305*::*Phep_4048 cat*; cross-complemented knock-out strain	This study
ATCC 43037 Δ*Tanf_07535*	Δ*Tanf_07535*::*ermF;* knock-out strain of *Tanf_07535*	This study
***Bacteroides fragilis* Strains**		
NCTC 9343	Type strain, wild-type	NCTC; [27]
NCTC 9343 Δ*BF4306*	Δ*BF4306*; knock-out strain of *BF4306*	[7]
***Pedobacter heparinus* strain**		
DSM 2366	Type strain, wild-type	DSMZ; [28]
**Plasmids**		
pJET1.2/blunt	Cloning vector; *amp*^R^	Thermo Fisher Scientific
pMAL_c2E	Expression vector, *amp*^R^	New England Biolabs
RK231	Broad-host-range mobilizing IncP plasmid, RK2 derivative; *kan*^R^	[29]
pCMF118	*E. coli*-*Bacteroides* shuttle vector, pFD340 derivative; *amp*^R^ *erm*^R^	[22]
pJET/TF0955ko	Template for amplifying the *erm* gene	[30]
pJET1.2/Δ*Tanf_01245*^+^	Template for amplifying the *cat* gene*; amp*^R^ *cat*^R^	[31]
pJET1.2/Δ*Tanf_01305*^+*BF4306*^	Cassette for reconstitution of Δ*Tanf_01305* with *BF4306*; *amp*^R^ *cat*^R^	This study
pJET1.2/Δ*Tanf_01305*^+*Phep_4048*^	Cassette for reconstitution of Δ*Tanf_01305* with *Phep_4048*; *amp*^R^ *cat*^R^	This study
pMT2	Cassette for reconstitution of Δ*BF4306* with *Tanf_01305*; *amp*^R^ *cat*^R^	This study
pMT21	Cassette for reconstitution of Δ*BF4306* with *Phep_4048*; *amp*^R^ *cat*^R^	This study
pJET1.2/Δ*Tanf_07535*	Δ*Tanf_07535*::*ermF; Tanf_07535* knock-out cassette; *amp*^R^ *erm*^R^	This study
pMAL_c2E/Tanf_01305	MBP-Tanf_01305 fusion; 72.5 kDa; *amp*^R^	This study
pMAL_c2E/BF4306	MBP-BF4306 fusion; 72.7 kDa; *amp*^R^	This study
pMAL_c2E/Phep_4048	MBP-Phep_4048 fusion; 72.0 kDa; *amp*^R^	This study

**Table 2:** ^1^H and ^13^C chemical shifts (ppm) and in parenthesis *J* couplings (Hz) of the nonasaccharide preparation from *B. fragilis* wild-type and the disaccharide glycopeptide from a *B. fragilis* Δ*BF4306* mutant.

Chemical Shift δ ^1^H (*J*_HH_) and ^13^C (*J*_CH_)								
Unit	1	2	3	4	5	6	CH_3_	CO
**A**	5.274 (4.0)	3.760 (4.0, 10.4)	3.707 (10.4, 3.1)	3.925 (3.1, 1.2)	4.261 (1.2, 5.5, 7.0)	3.719 (5.5, 11.5)		
α-Gal*p*						3.712 (7.0, 11.5)		
	102.87 (173.1)	71.26	72.14	72.14	74.30	64.24		
**B**	5.212 (4.1)	3.606 (4.1, 10.0)	3.828 (10.0, 8.8)	3.687 (8.8, 9.7)	4.205 (9.7)			
α-Glc*p*A	102.44 (171.6)	73.97	74.18	83.28	74.62	178.51		
**C**	5.145 (1.6)	3.639 (1.6, 3.5)	3.826 (3.5, 9.7)	3.364 (9.7, 9.7)	3.767 (9.7, 6.3)	1.248 (6.3)	3.436	
α-(2-O-Me)-Rha*p*	101.56 (168.4)	83.30	72.31	74.91	71.69	19.29	61.09	
**D**	5.044 (4.0)	4.122 (4.0, 11.2)	3.896 (11.2, 3.3)	3.930 (3.3, 0.8)	4.225 (0.8, 8.7, 3.5)	3.747 (8.7, 12.0)	2.010	
α-Gal*p*NAc						3.664 (3.5, 12.0)		
	102.32 (172.1)	52.51	70.21	71.50	74.04	64.58	24.80	177.24
**E**	4.842 (4.1)	3.819 (4.1, 10.3)	3.893 (10.3, 3.2)	4.042 (3.2, 0.8)	4.384 (0.8, 6.2)	1.147 (6.2)		
α-Fuc*p*	102.25 (172.2)	70.69	77.89	84.83	69.98	18.11		
**F**	4.814 (1.2)	4.741 (1.2, 3.3)	4.077 (3.3, 9.8)	4.059 (9.8, 9.3)	3.736 (9.3)		2.097	
β-Man*p*ANAc	103.23 (161.5)	55.70	78.46	78.84	79.89	177.72	24.78	177.61
**G**	4.601 (1.0)	3.914 (1.0, 3.7)	3.583 (3.7, 9.8)	3.482 (9.8, 9.7)	3.323 (9.7, 2.2, 7.0)	3.913 (2.2, 12.1)		
β-Man*p*						3.687 (7.0, 12.1)		
	102.27 (160.4)	73.51	75.36	69.44	79.57	63.70		
**H**	4.476 (8.0)	3.287 (8.0, 9.3)	3.568 (9.3, 8.8)	3.501 (8.8, 9.8)	3.529 (9.8, 3.5, 4.0)	3.911 (3.5, 11.4)		
β-Glc*p*						3.760 (4.0, 11.4)		
	104.77 (161.2)	76.39	76.80	79.09	77.99	62.42		
**I**	3.914	3.691	3.998	4.085	3.884	3.837		
Alditol	3.869					3.669		
	63.79	82.32	70.18	82.52	74.05	65.11		
**C’**	5.004 (1.5)	3.608 (1.5, 3.5)	3.770 (3.5, 9.4)	3.365 (9.4, 9.4)	3.956 (9.4, 6.3)	1.240 (6.3)	3.454	
α-(2-O-Me)-Rha*p*	100.49 (169.7)	83.13	72.68	74.89	71.61	19.15	61.30	
**I’**	4.877 (1.9)	3.941 (1.9, 3.7)	3.888 (3.7, 9.4)	3.768 (9.4, 9.2)	3.708 (9.2, 1.8, 5.2)	3.855 (1.8, 12.3)		
α-Man*p*						3.773 (5.2, 12.3)		
	103.09 (170.3)	72.55	71.65	78.58	74.76	63.27		
	A	β	γ		CO			
**Ser**	4.335 (3.9, 5.7)	4.181 (3.9, 11.5)						
		3.916 (5.7, 11.5)						
	55.69	68.56			n.d.			
**Val**	4.081 (6.1)	2.099 (6.1, 6.9)	0.934 (6.9)	0.903 (6.9)				
	55.75	33.10	21.56	20.07	n.d.			

n.d. not detected.

## Data Availability

The data presented in this study are available in the Supplementary Materials or upon request from C.S.

## Supplementary Materials

The following are available online at https://www.mdpi.com/article/10.3390/biom11121795/s1, Supplementary Methods: Preparation of *p*NP-α-d-Gal-Fuc As a Fucosidase Substrate. Table S1: Primers used in this study. Figure S1: Strategy for cross-complementation of a *T. forsythia* Δ*Tanf_01305* knock-out mutant with the homologous genes *BF4306* from *B. fragilis* and *Phep_4048* from *P. heparinus*, respectively, and confirmation by screening PCR. (**A**) The genomic organization of the Tanf_01305 locus is shown for the parent strain *T. forsythia* ATCC 43037, the Δ*Tanf_01305* mutant and the cross-complemented mutants Δ*Tanf_01305^+BF4306^* and Δ*Tanf_01305^+Phep_4048^*. Black coloured arrows represent primers used for PCR amplification of genes and homologous regions, red coloured primers represent those used to screen for correct integration of the cross-complementation cassettes; restriction sites used for cloning are indicated (not drawn to scale). (**B**) Agarose gel electrophoresis (left) confirms the successful integration of *BF4306* at the *Tanf_01305* locus using the screening primers 494/JB_23 (1888 bp) and 48/495 (1141 bp) when using genomic DNA of the Δ*Tanf_01305^+BF4306^* strain. Screening primers 494/495 yield in a 3649-bp PCR product on genomic DNA of the cross-complemented mutant Δ*Tanf_01305^+BF4306^*, whereas the same primer pair results in a 3023-bp product on genomic DNA of the *T. forsythia* wild-type. The successful integration of *Phep_4048* at the *Tanf_01305* locus was likewise confirmed using the screening primers 494/JB_25 (1867 bp) and 48/495 (1137 bp) when using genomic DNA of the Δ*Tanf_01305^+Phep4048^* strain (right). Screening primers 494/495 yield in a 3630-bp PCR product from genomic DNA of the cross-complemented mutant Δ*Tanf_01305^+Phep_4048^*, whereas the same primer pair results in a 3023-bp product from genomic DNA of the *T. forsythia* wild-type. O’Gene Ruler 1 kb Plus DNA Ladder (Thermo Fisher Scientific) was used as a gene ladder). Figure S2: Strategy for the generation of a *T. forsythia* ATCC 43037 *fcl* deficient mutant and confirmation by PCR. (**A**) The genomic organization of the *Tanf_07535* locus is shown for the parent strain *T. forsythia* ATCC 43037 and the Δ*Tanf_07535* mutant. Black coloured arrows represent primers used for PCR amplification of genes and homologous regions, red coloured primers represent those used to screen for correct integration of the knock-out (not drawn to scale). (**B**) Agarose gel electrophoresis confirms the deletion of *Tanf_07535* using the up-stream primers 532/524 (1212 bp) and down-stream primers 525/533 (1085 bp) from genomic DNA of *T. forsythia* ATCC 43037 Δ*Tanf_07535* mutant with integrated *erm*. Primers 534/535 yield in a 7898-bp PCR fragment when using *T. forsythia* wild-type genomic DNA, whereas this fragment is absent from genomic DNA of the Δ*Tanf_07535* mutant confirming the loss of the gene (log); O´Gene Ruler 1 kb Plus DNA Ladder (Thermo Fisher Scientific) was used as a gene ladder. Figure S3: Amino acid sequences of rFucT-MBP chimera used in this study. Sequences are color-coded as follows: MBP (maltose binding protein); linker sequence; rFucT. Figure S4: ^1^H NMR spectra of the *B. fragilis* *O*-glycan released from glycopeptides by reductive β-elimination. The bottom trace shows the conventional ^1^H NMR, whereas in the top trace, the residual solvent signal HDO at 4.7 ppm was reduced significantly by applying a 100 ms diffusion filter. Figure S5: 2D-HSQC spectrum of the *B. fragilis* *O*-glycan released from glycopeptides by reductive β-elimination correlating the carbon signals (left projection) with the directly attached protons (top projection) over one bond. The insert shows the expanded region for the anomeric cross-peaks. The sugar units **A**–**H** are denoted on the corresponding ^1^H NMR signals in the top trace. Figure S6: Structure elucidated by 700 MHz NMR spectroscopy of the *B. fragilis* wild-type nonasaccharide obtained after reductive β-elimination. While the biochemical data are in full support of the presence of l-fucose, the absolute configuration of the rhamnose unit C remains to be firmly established and has only been tentatively assigned to l-configuration. Figure S7: ^1^H NMR spectra of three glycopeptides derived from a *B. fragilis* Δ*BF4306* mutant. The bottom trace is the spectrum from preparation f28, the middle trace from f32, and the top trace from f36. The residual solvent signal HDO (at 4.7 ppm) was suppressed by using presaturation for 3 s. The expansions on the right side show the anomeric regions. Preparation f28 was used for further NMR investigations as essentially only two intense anomeric signals for the disaccharide peptide show up. Preparations f32 and f36 show more signals due to heterogeneity of the peptide part. Figure S8: Simulated and experimental ^1^H NMR spectra from the *B. fragilis* Δ*BF4306* glycopeptide f28. The top trace shows the experimental spectrum with an expansion as insert for the aliphatic methyl region. Below are two traces with the simulated spin systems of the two sugar units **C’** and **I’**, the assignment of the individual protons is denoted at the corresponding signals. Trace **C’** shows an expansion as insert showing the methyl group of the Rha. Figure S9: SDS-PAGE analysis of purified FucTs used in this study. Recombinantly expressed MBP chimera (rTanf_01305-MBP; calculated molecular weight: 72.5 kDa; rBF4306-MBP; calculated molecular weight: 72.7 kDa; rPhep_4048-MBP, calculated molecular weight: 72.0 kDa) were purified via an amylose resin (GE Healthcare; running buffer: 20 mM Tris-HCl pH 7.5, 200 mM NaCl; elution buffer: 20 mM Tris-HCl, pH 7.5, 200 mM NaCl, 10 mM maltose). 10 µg of recombinant enzyme was loaded per lane. Proteins were stained with CBB after separation on a 7.5% SDS-PA gel. PageRuler Plus Prestained Protein Ladder (Thermo Fisher Scientific) was used as a protein molecular weight marker. Figure S10: In vitro fucosyltransferase activity assays with other *p*NP-sugar substrates. Developed TLC plates show no formation of (**A**) *p*NP-α-d-Xyl-Fuc, (**B**) *p*NP-α-d-GlcA-Fuc or (**C**) *p*NP-β-d-GlcNAc-Fuc when using recombinant FucT from *T. forsythia* (rTanf_01305), *B. fragilis* (rBF4306) and *P. heparinus* (rPhep_4048), respectively. Figure S11: Fucosidase treatment of *p*NP-Gal-Fuc generated through Tanf_01305 activity on *p*NP-Gal. Commercially available fucosidases α1,2 fucosidase, α1,3/4 fucosidase and α1,2/4/6 fucosidase O as well as *T. forsythia* fucosidase rTfFuc1 were tested for their ability to cleave Fuc from *p*NP-α-Gal-Fuc. Reactions were analyzed by TLC on silica gel 60 F_254_ plates. Fucosidase O and rTfFuc1 were active on *p*NP-α-Gal-Fuc.

## Abbreviations

ACN, acetonitrile; BHI, brain heart infusion; cat, chloramphenicol; CBB, Coomassie Brilliant Blue; CID, collision-induced dissociation; COSY, Correlation Spectroscopy; DQF, double quantum filtered; erm, erythromycin; ESI-MS, electrospray ionization mass spectrometry; Fcl, GDP-l-fucose synthase; Fuc, fucose; FucT, fucosyltransferase; Gal, galactose; GDP, guanosine diphosphate; Glc, glucose; GlcA, glucuronic acid; GlcNAc, *N*-acetylglucosamine; GT, glycosyltransferase; HMBC, Heteronuclear Multiple Bond Correlation Spectroscopy; HSQC, Heteronuclear Single Quantum Coherence Spectroscopy; IPTG, isopropyl-β-d-1-thiogalactopyranoside; LB medium, Luria Bertani medium; LC, liquid chromatography; *lfg*, locus for glycosylation; LPS, lipopolysaccharide; Man, mannose; MBP, maltose binding protein; Man*p*ANAc, 2-*N*-acetylamino-2-deoxy-uronic acid; MS, mass spectrometry; NOESY, Nuclear Overhauser Effect Spectroscopy; PGC, porous graphitized carbon; *p*NP, *para*-nitrophenylphosphate; r, recombinant; TDP, thymidine diphosphate; TfsA/B, *Tannerella forsythia* surface-layer protein A/B; TOCSY, Total Correlation Spectroscopy; TLC, thin-layer chromatography; Xyl, xylose.
