# Supplementary material for: A Combination of Structural, Genetic, Phenotypic and Enzymatic Analyses Reveals the Importance of a Predicted Fucosyltransferase to Protein O-Glycosylation in the Bacteroidetes
Source: Biomolecules. 2021 Nov 30;11(12):1795. doi: 10.3390/biom11121795 (PMC8698959; doi:10.3390/biom11121795)
Supplement: Supplementary file 1 [file biomolecules-11-01795-s001.zip › biomolecules-1446556-supplementary.pdf]

biomolecules

Supplementary Materials

A Combination of Structural, Genetic, Phenotypic and Enzymatic Analyses Reveals the Importance of a Predicted Fucosyltransferase to Protein *O*-Glycosylation in the Bacteroidetes

Markus B. Tomek [1,†], Bettina Janesch [1,†], Matthias L. Braun [1,†], Manfred Taschner [1], Rudolf Figl [2], Clemens Grünwald-Gruber [2], Michael J. Coyne [3], Markus Blaukopf [4], Friedrich Altmann [2], Paul Kosma [4], Hanspeter Kählig [5], Laurie E. Comstock [3] and Christina Schäffer [1,*]

[1] *NanoGlycobiology* Unit, Institute of Biologically Inspired Materials, Department of NanoBiotechnology, Universität für Bodenkultur Wien, Muthgasse 11, A-1190 Vienna, Austria; markus.tomek@boku.ac.at (M.B.T.); bettina.janesch@gmx.at (B.J.); math.b@gmx.at (M.L.B.); taschner@lifetaq.com (M.T.)
[2] Institute of Biochemistry, Department of Chemistry, Universität für Bodenkultur Wien, Muthgasse 18, A-1190 Vienna, Austria; rudolf.figl@boku.ac.at (R.F.); clemens.gruber@boku.ac.at (C.G.-G.); friedrich.altmann@boku.ac.at (F.A.)
[3] Department of Microbiology and the Duchossois Family Institute, University of Chicago, KCBD, 900 E. 57th Street, Chicago, IL 60637, USA; mcoyne@uchicago.edu (M.J.C.); lecomstock@uchicago.edu (L.E.C.)
[4] Institute of Organic Chemistry, Department of Chemistry, Universität für Bodenkultur Wien, Muthgasse 18, A-1190 Vienna, Austria; markus.blaukopf@boku.ac.at (M.B.); paul.kosma@boku.ac.at (P.K.)
[5] Department of Organic Chemistry, Faculty of Chemistry, University of Vienna, Währinger Strasse 38, A-1090 Vienna, Austria; hanspeter.kaehlig@univie.ac.at
* Correspondence: christina.schaeffer@boku.ac.at; Tel.: +43-1-47654-80203
† These authors contributed equally to this work.

Supplementary Methods

Preparation of pNP-α-D-Gal-Fuc As a Fucosidase Substrate

For an indirect determination of the L-Fuc-Gal linkage in the *T. forsythia O*-glycan structure, the *p*NP-α-L-Gal-Fuc product obtained from the in vitro rTanf_01305 reaction was used. For that purpose, the reaction mixture was layered onto a preparative glass-backed TLC plate (TLC Silica gel 60 G F254 glass plates, Merck Millipore) and allowed to dry completely. The TLC plate was developed using an ACN:H_2O (17:2) solvent mixture. Once dry, the product band of interest was carefully scraped off with a spatula and transferred into a 1.5-mL reaction tube. The silica was washed twice with 100 µL of ACN:H_2O (17:2) and the supernatants were collected. Remaining silica was removed by centrifugation (16,000× *g*, 5 min, 22 °C) and samples were dried at 70 °C. Purity was checked on a TLC plate; the washing process was repeated until no residual *p*NP-Gal was visible.

The following fucosidases were tested for activity on *p*NP-α-L-Gal-Fuc:α1,2 fucosidase (catalyses the hydrolysis of linear α1,2-linked Fuc), α1,3/4 fucosidase (catalysis the hydrolysis of terminal, non-reducing α1,3 and α1,4-linked Fuc) and α1,2/4/6 fucosidase O (catalyses the hydrolysis of terminal α1,2, α1,4 and α1,6-linked Fuc with the highest efficiency on α1,6 linked Fuc residues) (all from New England Biolabs) as well as rTfFuc1 (a *T. forsythia* α1,2 fucosidase with additional α1,6 specificity on small unbranched substrates) [42].

The *p*NP-α-D-Gal-Fuc product was resuspended in 20 µL of ultrapure water (Milli-Q). Standard assay conditions were performed in 10 µL at 37 °C for 2 h were: 4 µL of *p*NP-α-D-Gal Fuc, 2 µL of fucosidase, 1× GlycoBuffer 1 (New England Biolabs), supplemented with 100 µg/mL BSA. For the rTfFuc1, 4 µL (approximately 1.6 µg) was used for hydrolysis. TLC was carried out on silica plates as described above.

Supplementary Table

Table S1. Primers used in this study.

Primer name	Primer Sequence (5′–3′)
516	gtacGAATTCATGAGTCCGCTGTTTAGCATTATTAC
517	gtacAAGCTTTTAATAGTTGTTGCCAAACAG
JB_172	aatcaGAATTCATGACAGCAGCCAAACCCACC
JB_173	aatcaGGATCCTTAATCGTTGGTTTTTCCGTGC
JB_174	aatcaGAATTCATGCATAGCCATCCCAGTCC
JB_175	aatcaAAGCTTTCAGAAGGGTGCATCGGAAG
**Cross-complementations**	
48 [a]	GTCAGATAGGCCTAATGACTGGC
490 [b]	GGGATATCGTATTGACCGGCATCCTC
494 [b]	CAGCCTAAGTGGTTCGGCAAGTTC
495 [b]	CATCGTTCGGGCCCTTGCACGATAAGC
JB_6	**GAATTTGGGACTGGGATGGCTATGCAT**ATATTATGACAATAGACTGTGATATAATG
JB_7	**CAGTCTATTGTCATAATAT**ATGCATAGCCATCCCAGTCCCAAATTC
JB_10	**CTTACATTATAATGTCGATTCA**TTATAAAAGCCAGTCATTAG
JB_11	**GGCCTAATGACTGGCTTTTATAA**TGAATCGACATTATAATGTAAG
JB_13	**ACGGATAAGGTGGGTTTGGCTGCTGTCAT**ATATTATGACAATAGACTGTGATATAATG
JB_14	**CAGTCTATTGTCATAATAT**ATGACAGCAGCCAAACCCACCTTATCC
JB_19	gctaGAGCTCATCGTAATGCGAGCTGGCTCCGTAG
JB_23	GAGCTCgtacGGTACCTCAGAAGGGTGCATCGGAAGGAGTAGC
JB_24	gctaGGTACCATGAACTTTAATAAAATTGATTTAGAC
JB_25	GAGCTCtgacGGTACCTTAATCGTTGGTTTTTCCGTGCCTG
Phep_4048_F	aaccGGATCCCCAGACAATATACAGACCTCTACCAA
Phep_4048_R	ccttGGATCCAAAAATTTAATCGTTGGTTTTTCC
Tanf_01305_F	gatcGGATCCGTCTATTGTCATAATATATGTCTCCTTTGTTTTCC
Tanf_01305_R	aagctGGATCCCTAATAATTGTTCCCGAAAAGCTTCGCC
**GDP-L-Fuc synthase knock-out**	

460	ATGACAAAAAAGAAATTGCCCGTTCGTTTTAC
461	CTACGAAGGATGAAATTTTTCAGGGACAAC
486	**CGAACGGGCAATTTCTTTTTTGTCAT**GAGTAGCGTTCAGTTTTCAGTATTG
487	ATACGGGACGGATCGAGCATTTG
488	GTCCGTTCTCAATCTTCACATCAG
489	**GTCCCTGAAAAATTTCATCCTTCGTAG**CTCTCCCATCGTTCCGAGCTCCTG
524	GTAAAACGAACGGGCAATTTCTTTTTTGTCAT
525	CCCTGAAAAATTTCATCCTTCGTAG
532	CCGCTGTCTGCTTGGAAGACG
533	CGAAACGCACATGTTTCCTACCC
534	GTCGGCCATTTGGAGCAACC
535	TGAAGGGTCGAAGGCAAGTG

Nucleotides used for overlap-extension PCRs are written in bold, artificial restriction sites are underscored. Lowercase letters indicate artificially introduced bases to improve restriction enzyme digestion.[a] [31] [b] [10].

Supplementary Figures

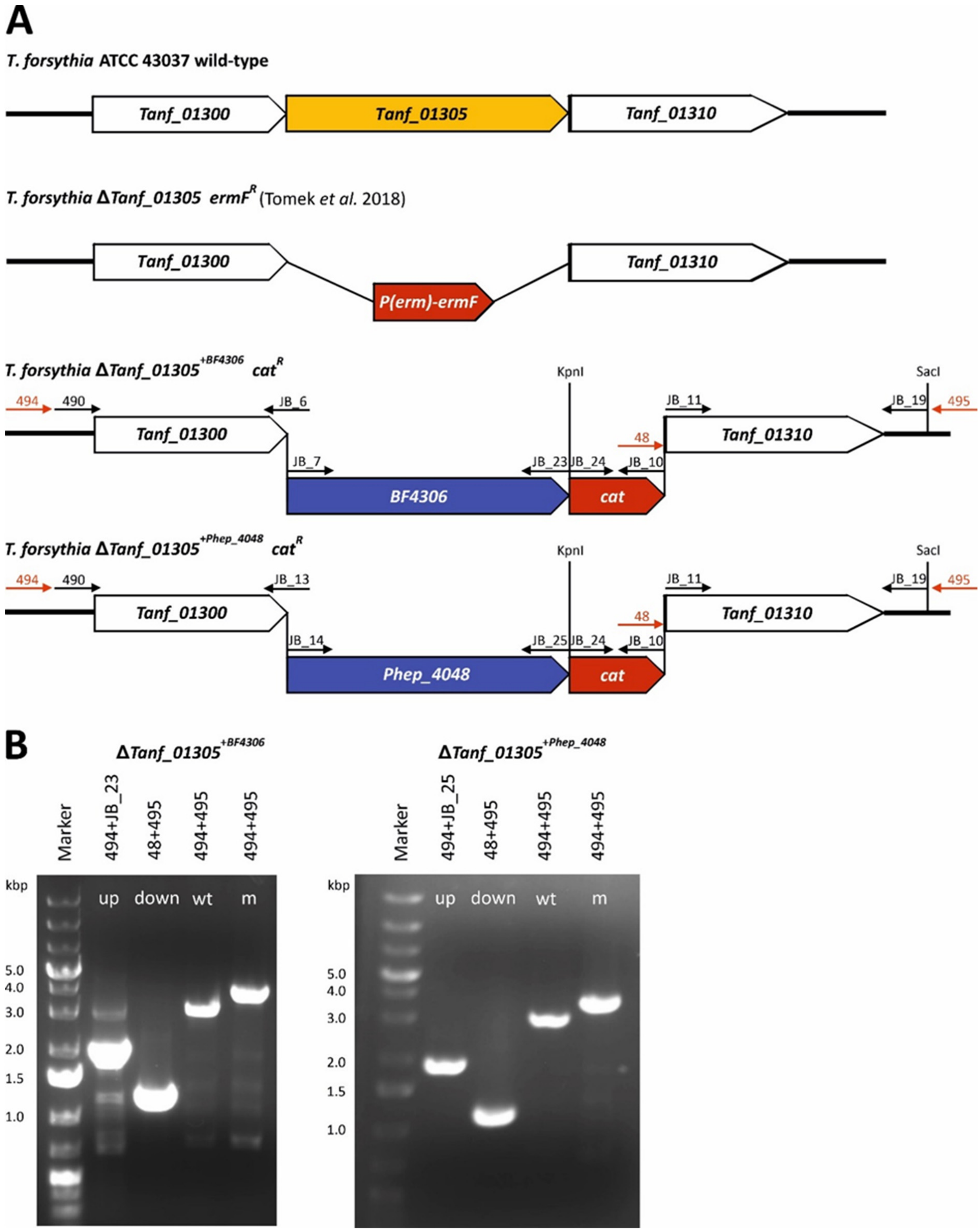

Figure S1. Strategy for cross-complementation of a *T. forsythia* Δ*Tanf_01305* knock-out mutant with the homologous genes *BF4306* from *B. fragilis* and *Phep_4048* from *P. heparinus*, respectively, and confirmation by screening PCR. (**A**) The genomic organization of the *Tanf_01305* locus is shown for the parent strain *T. forsythia* ATCC 43037, the Δ*Tanf_01305* mutant [10] and the cross-complemented mutants Δ*Tanf_01305*$^{+BF4306}$ and Δ*Tanf_01305*$^{+Phep\_4048}$. Black coloured arrows represent primers used for PCR amplification of genes and homologous regions, red coloured primers represent those used to screen for correct integration of the cross-complementation cassettes; restriction sites used for cloning are indicated (not drawn to scale). **(B)** Agarose gel electrophoresis (left) confirms the successful integration of *BF4306* at the *Tanf_01305* locus using the screening primers 494/JB_23 (1888 bp) and 48/495 (1141 bp) when using genomic DNA of the Δ*Tanf_01305*$^{+BF4306}$ strain. Screening primers 494/495 yield in a

3649-bp PCR product from genomic DNA of the cross-complemented mutant Δ*Tanf_01305*$^{+BF4306}$, whereas the same primer pair results in a 3023-bp product from genomic DNA of the *T. forsythia* wild-type. The successful integration of *Phep_4048* at the *Tanf_01305* locus was likewise confirmed using the screening primers 494/JB_25 (1867 bp) and 48/495 (1137 bp) when using genomic DNA of the Δ*Tanf_01305*$^{+Phep4048}$ strain (right). Screening primers 494/495 yield in a 3630-bp PCR product from genomic DNA of the cross-complemented mutant Δ*Tanf_01305*$^{+Phep\_4048}$, whereas the same primer pair results in a 3023-bp product from genomic DNA of the *T. forsythia* wild-type. O´Gene Ruler 1 kb Plus DNA Ladder (Thermo Fisher Scientific) was used as a gene ladder.

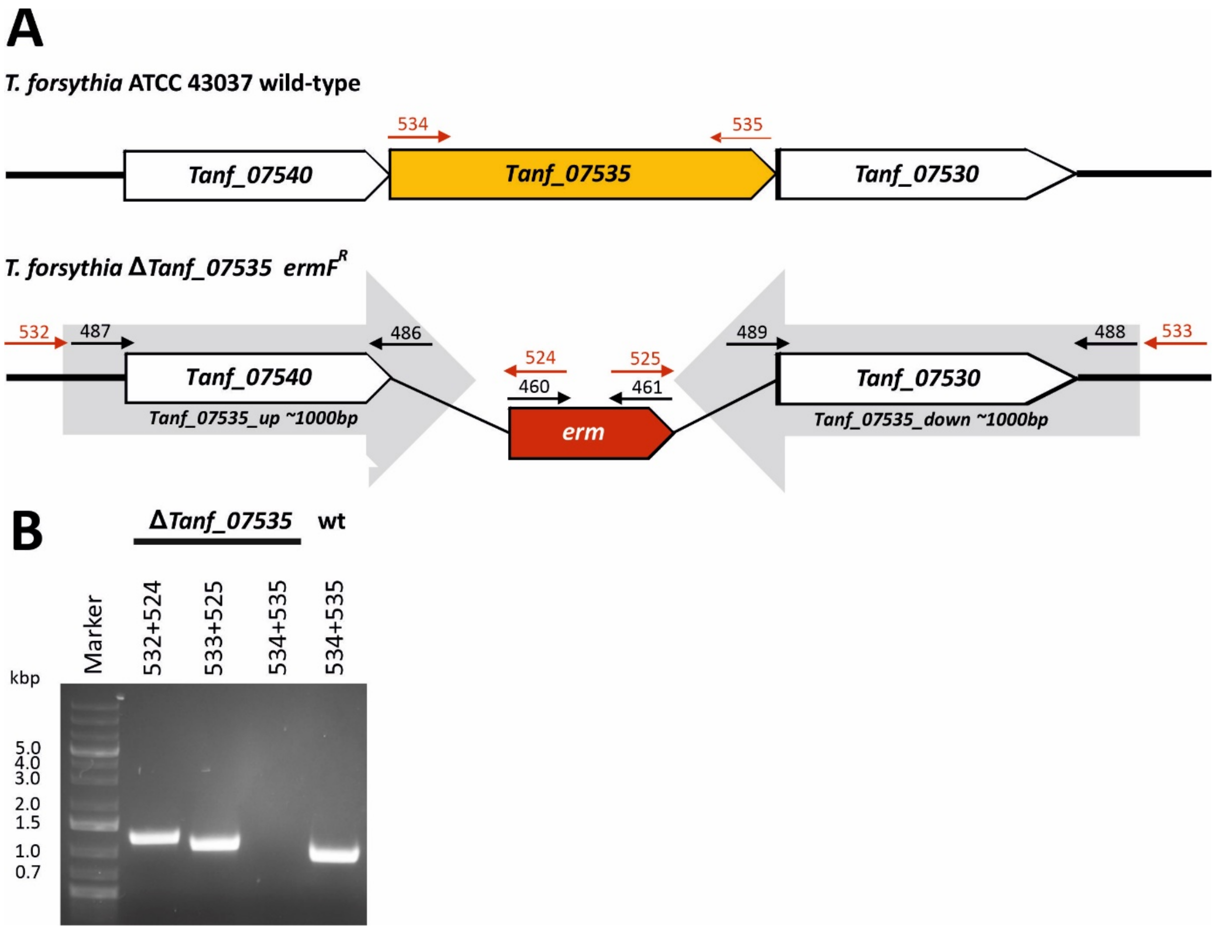

Figure S2. Strategy for the generation of a *T. forsythia* ATCC 43037 *fcl* deficient mutant and confirmation by PCR. (**A**) The genomic organization of the *Tanf_07535* locus is shown for the parent strain *T. forsythia* ATCC 43037 and the Δ*Tanf_07535* mutant. Black coloured arrows represent primers used for PCR amplification of genes and homologous regions, red coloured primers represent those used to screen for correct integration of the knock-out (not drawn to scale). (**B**) Agarose gel electrophoresis confirms the deletion of *Tanf_07535* using the up-stream primers 532/524 (1212 bp) and down-stream primers 525/533 (1085 bp) from genomic DNA of *T. forsythia* ATCC 43037 Δ*Tanf_07535* mutant with integrated *erm*. Primers 534/535 yield in a 7898-bp PCR fragment when using *T. forsythia* wild-type genomic DNA, whereas this fragment is absent from genomic DNA of the Δ*Tanf_07535* mutant confirming the loss of the gene (log); O´Gene Ruler 1 kb Plus DNA Ladder (Thermo Fisher Scientific) was used as a gene ladder.

MBP-Tanf_01305 chimera

MKTEEGKLVIWINGDKGYNGLAEVGKKFEKDTGIKVTVEHPDKLEEKFPQVAATGDGPDIIFW
AHDRFGGYAQSGLLAEITPDKAFQDKLYPFTWDAVRYNGKLIAYPIAVEALSLIYNKDLLPNPP
KTWEEIPALDKELKAKGKSALMFNLQEPYFTWPLIAADGGYAFKYENGKYDIKDVGVDNAGA
KAGLTFLVDLIKNKHMNADTDYSIAEAAFNKGETAMTINGPWAWSNIDTSKVNYGVTVLPTFK
GQPSKPFVGVLSAGINAASPNKELAKEFLENYLLTDEGLEAVNKDKPLGAVALKSYEEELAKDP
RIAATMENAQKGEIMPNIPQMSAFWYAVRTAVINAASGRQTVDEALKDAQTNSSSNNNNNN
NNNNLGDDDDKVPEFMSPLFSIITVTYNAEKVLERTLRSVAEQSYARIEYIVIDGASTDGTMEIVT
SYKSQSPGLVRFEVISEPDKGLYDAMNKGLRVATGDYVWFLNAGDTLQSPETVARLTEVAERN
GMPDILYGETDVVDEQGKFLAARRLKAPEHLTWKSFCTGMLVCHQAFVMKREAAPEYDLQYR
FSADFDWCIRCMKQSTAIVNSHLRLIHYLAEGMTTRNRKASLKERYRIMCNYYGTFPTRIRHLW
FALRFYWAKLFGNNY

MBP-BF4306 chimera

MKTEEGKLVIWINGDKGYNGLAEVGKKFEKDTGIKVTVEHPDKLEEKFPQVAATGDGPDIIFW
AHDRFGGYAQSGLLAEITPDKAFQDKLYPFTWDAVRYNGKLIAYPIAVEALSLIYNKDLLPNPP
KTWEEIPALDKELKAKGKSALMFNLQEPYFTWPLIAADGGYAFKYENGKYDIKDVGVDNAGA
KAGLTFLVDLIKNKHMNADTDYSIAEAAFNKGETAMTINGPWAWSNIDTSKVNYGVTVLPTFK
GQPSKPFVGVLSAGINAASPNKELAKEFLENYLLTDEGLEAVNKDKPLGAVALKSYEEELAKDP
RIAATMENAQKGEIMPNIPQMSAFWYAVRTAVINAASGRQTVDEALKDAQTNSSSNNNNNN
NNNNLGDDDDKVPEFMHSHPSPKFSVITVTYNAEKVLEDTVQSVISQTYHHVEYIIIDGASKDG
TLEIVNRYRNRINQLVSEPDKGLYDAMNKGIALATGDYLCFLNAGDSFHEDDTLQKMVHSING
NELPDILYGETALVDAERHFLRMRRLSAPETLNWKSFKQGMLVCHQAFFPRHTLIEPYDLQYRF
SADFDWCIHIMKKARTFHNTHLILIDYLAEGMTTQNHKASLLERFRIMTRHYGLLSTLAHHAW
FVVRSFSRNSATPSDAPF

MBP-Phep_4048 chimera

MKTEEGKLVIWINGDKGYNGLAEVGKKFEKDTGIKVTVEHPDKLEEKFPQVAATGDGPDIIFW
AHDRFGGYAQSGLLAEITPDKAFQDKLYPFTWDAVRYNGKLIAYPIAVEALSLIYNKDLLPNPP
KTWEEIPALDKELKAKGKSALMFNLQEPYFTWPLIAADGGYAFKYENGKYDIKDVGVDNAGA
KAGLTFLVDLIKNKHMNADTDYSIAEAAFNKGETAMTINGPWAWSNIDTSKVNYGVTVLPTFK
GQPSKPFVGVLSAGINAASPNKELAKEFLENYLLTDEGLEAVNKDKPLGAVALKSYEEELAKDP
RIAATMENAQKGEIMPNIPQMSAFWYAVRTAVINAASGRQTVDEALKDAQTNSSSNNNNNN
NNNNLGDDDDKVPEFMTAAKPTLSVITVVYNNVKDIERTMLSVLNQTYPNIEYLLIDGGSTDGT
KDIIYKYKSRLAQFISEPDKGIYDAMNKGLALATGNYVLFMNSGDELYASDTVAEVFDTASSADI
YYGETEMYNEKWESLGQRRHCSPENFNWKSFRYGMSISHQAIYVKRSLAAPFDLRYKYSADID
WIIKAAKKASSIVNTHMYVAKYLVGGMSKKKHLASLKERFRIFTKYYGLVPNLINHIFISFNLVQ
YYFRHGKTND

Figure S3. Amino acid sequences of rFucT-MBP chimera used in this study. Sequences are color-coded as follows: MBP (maltose binding protein) (green); linker sequence (blue); rFucT.

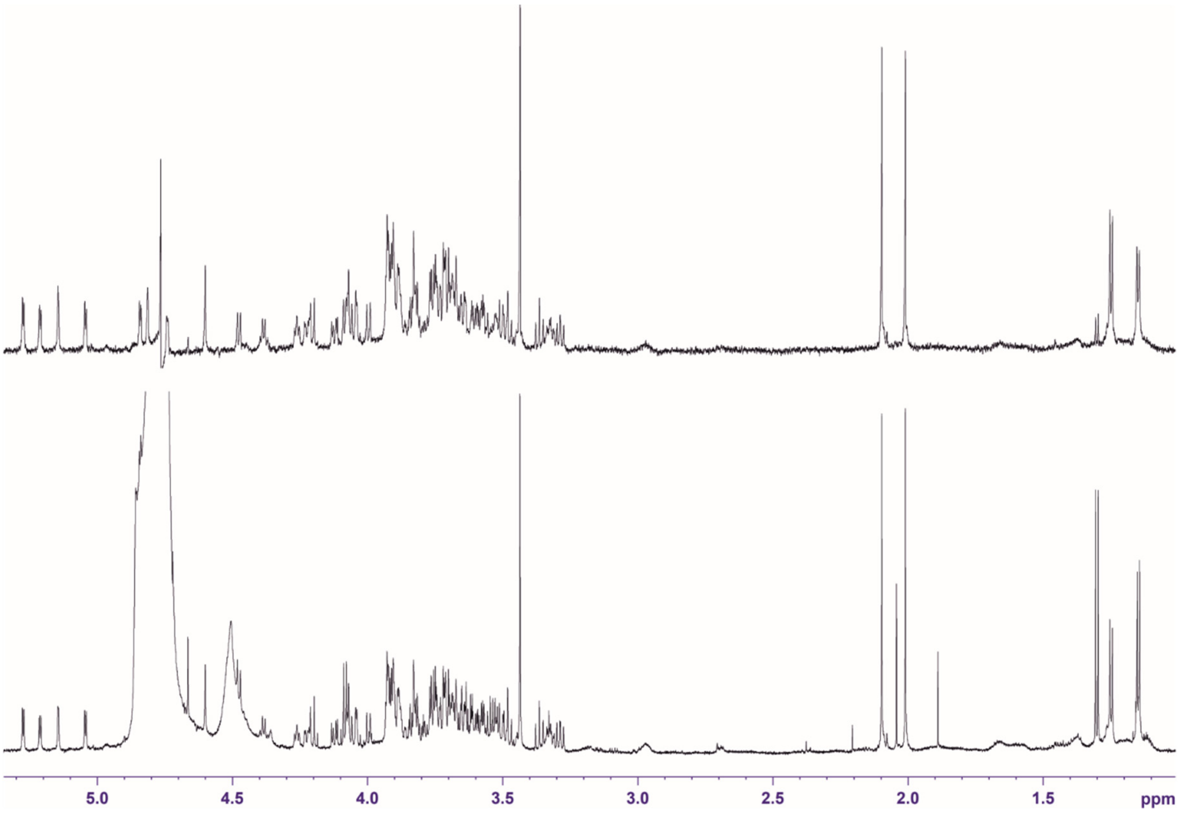

Figure S4. ^{1}H NMR spectra of the *B. fragilis O*-glycan released from glycopeptides by reductive β-elimination. The bottom trace shows the conventional ^{1}H NMR, whereas in the top trace the residual solvent signal HDO at 4.7 ppm was reduced significantly by applying a 100 ms diffusion filter.

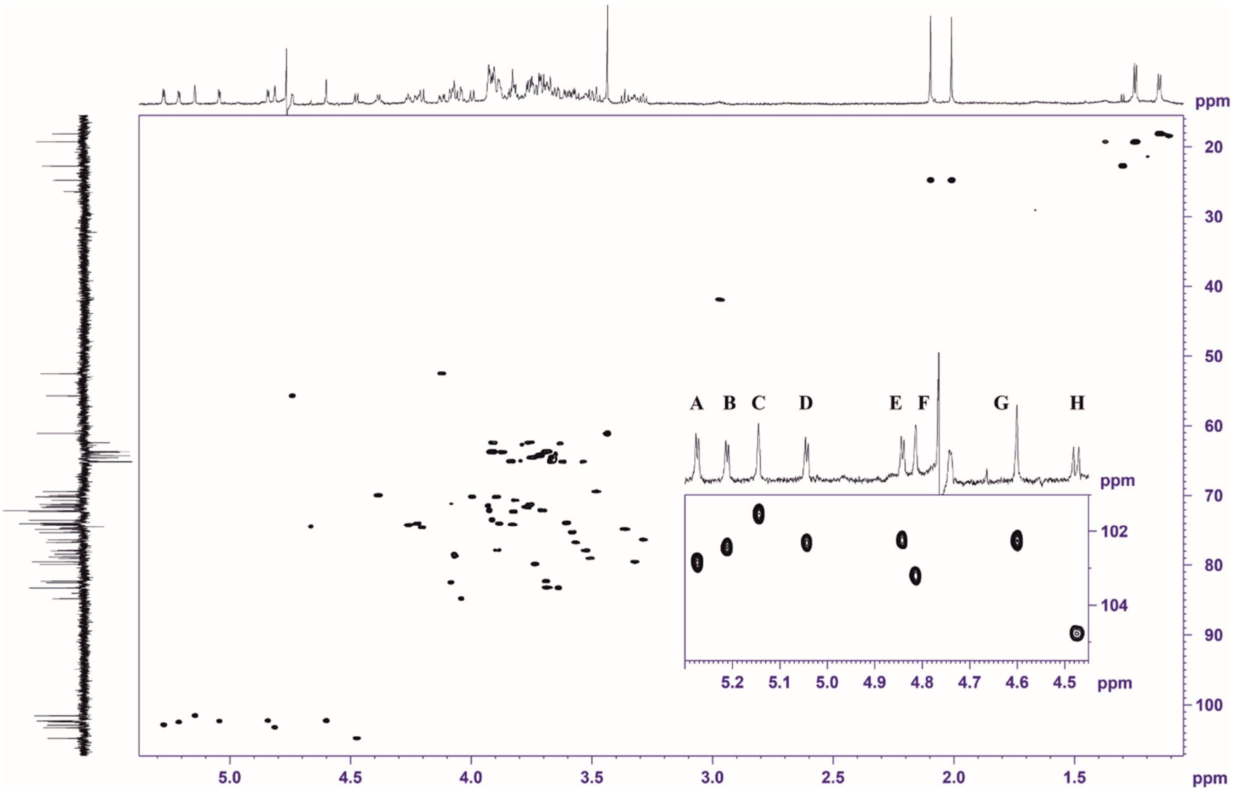

Figure S5. 2D-HSQC spectrum of the *B. fragilis O*-glycan released from glycopeptides by reductive β-elimination correlating the carbon signals (left projection) with the directly attached protons (top projection) over one bond. The insert shows the expanded region for the anomeric cross-peaks. The sugar units **A**–**H** are denoted on the corresponding ^{1}H NMR signals in the top trace.

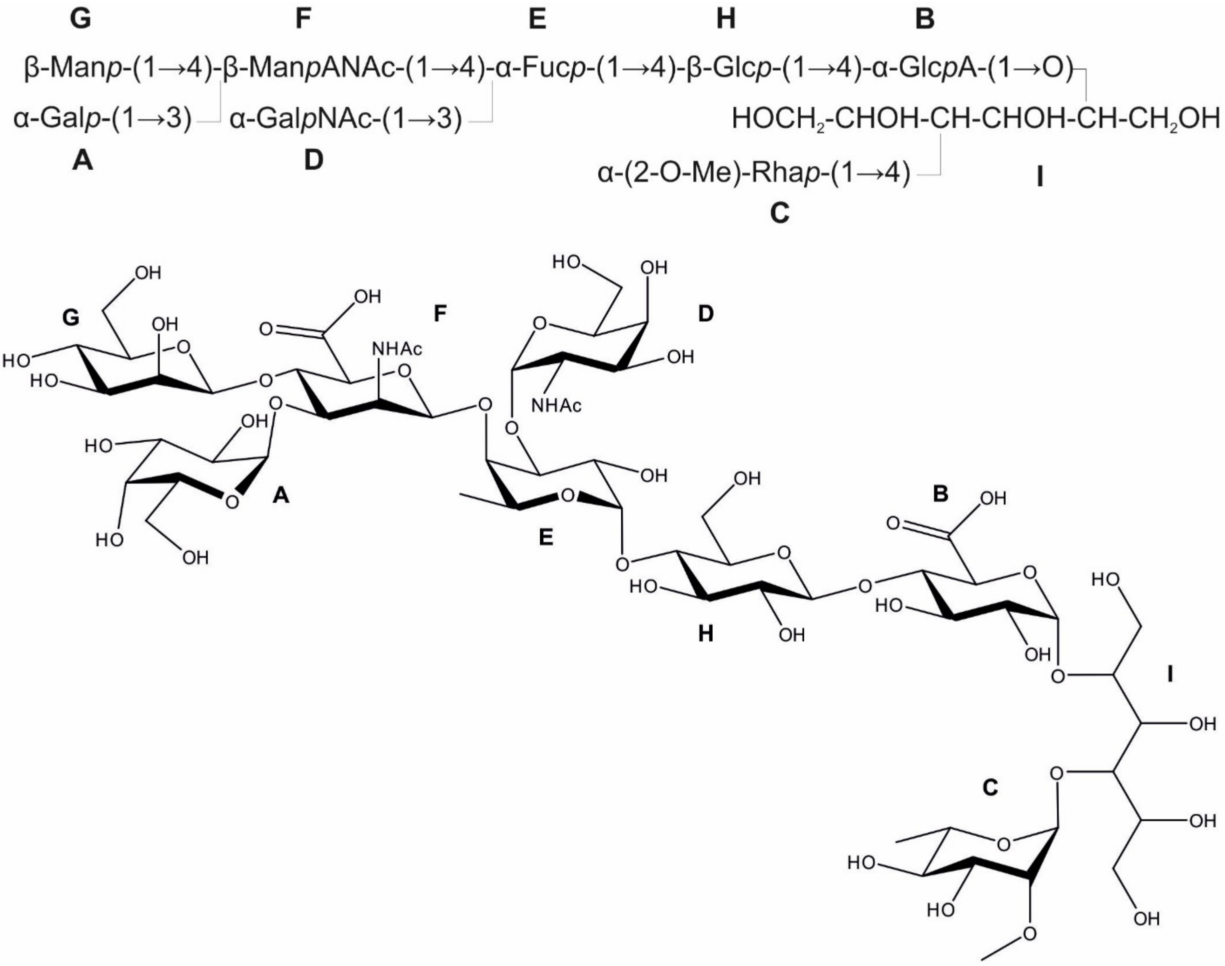

Figure S6. Structure elucidated by 700 MHz NMR spectroscopy of the *B. fragilis* wild-type nonasaccharide obtained after reductive β-elimination. While the biochemical data are in full support of the presence of L-fucose, the absolute configuration of the rhamnose unit **C** remains to be firmly established and has only been tentatively assigned to L-configuration.

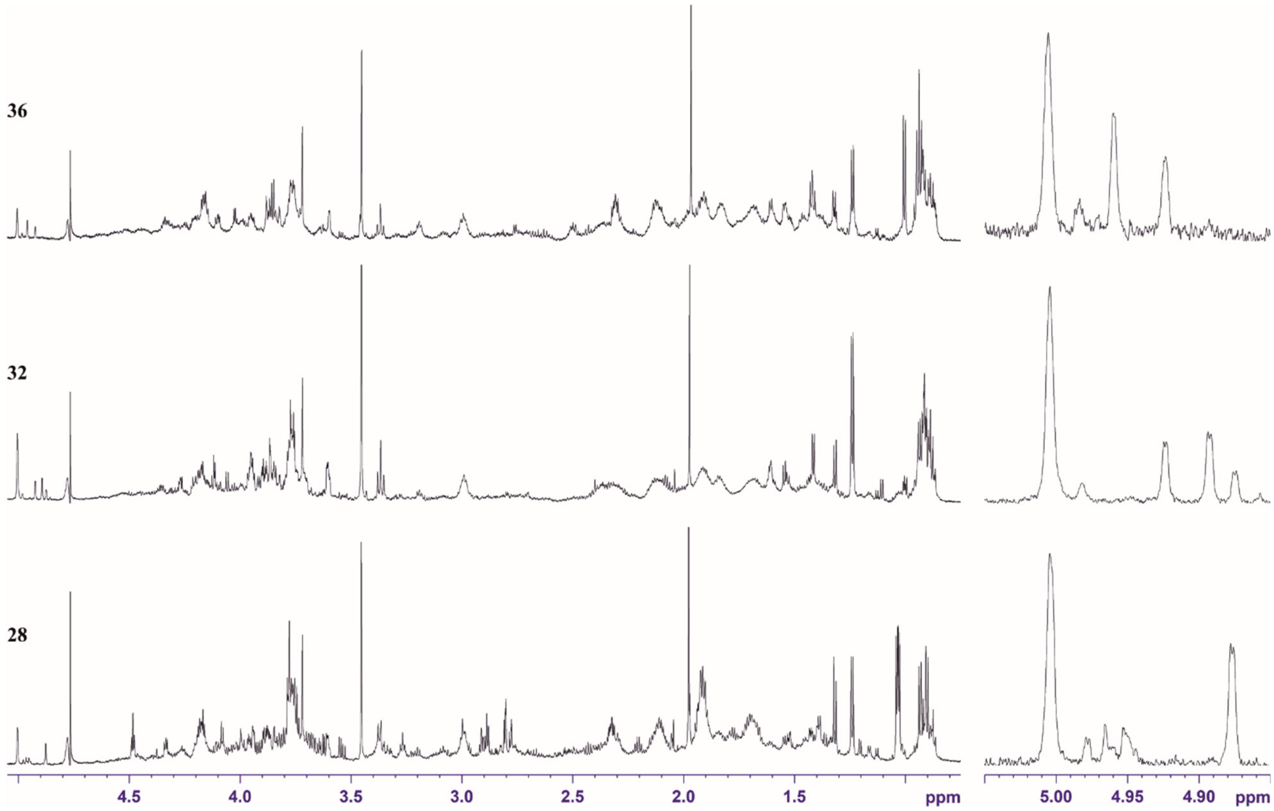

Figure S7. ^{1}H NMR spectra () of three glycopeptides derived from a *B. fragilis ΔBF4306* mutant. The bottom trace is the spectrum from preparation f28, the middle trace from f32, and the top trace from f36. The residual solvent signal HDO (at 4.7 ppm) was suppressed by using presaturation for 3 s. The expansions on the right side show the anomeric regions. Preparation f28 was used for further NMR investigations as essentially only two intense anomeric signals for the disaccharide peptide show up. Preparations f32 and f36 show more signals due to heterogeneity in the peptide part.

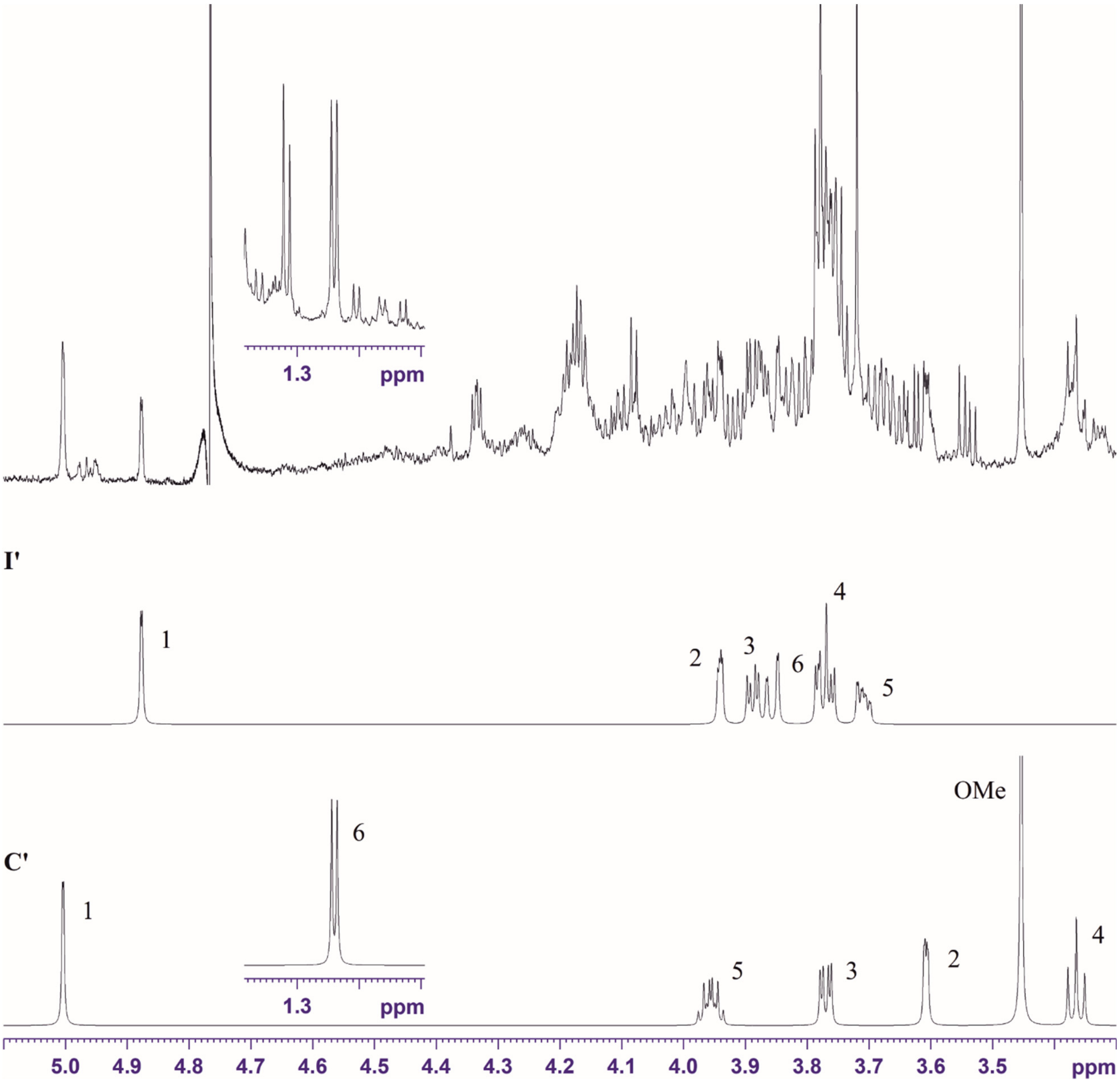

Figure S8. Simulated and experimental ^{1}H NMR spectra from the *B. fragilis ΔBF4306* glycopeptide f28. The top trace shows the experimental spectrum with an expansion as insert for the aliphatic methyl region. Below are two traces with the simulated spin systems of the two sugar units **C′** and **I′**, the assignment of the individual protons is denoted at the corresponding signals. Trace **C′** shows an expansion as insert showing the methyl group of the Rha.

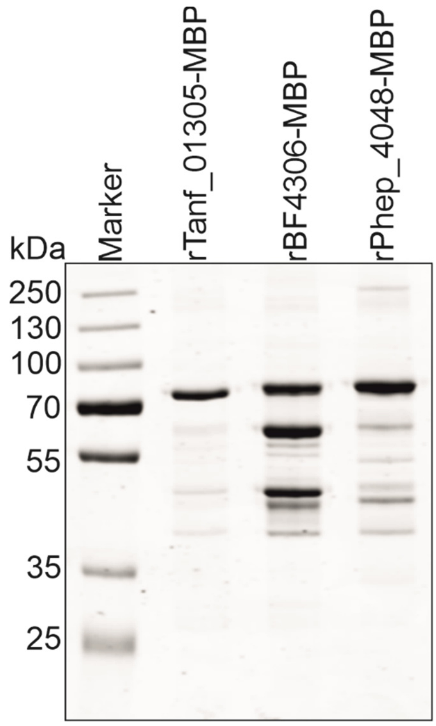

Figure S9. SDS-PAGE analysis of purified FucTs used in this study. Recombinantly expressed MBP chimera (rTanf_01305-MBP; calculated molecular weight: 72.5 kDa; rBF4306-MBP; calculated molecular weight: 72.7 kDa; rPhep_4048-MBP, calculated molecular weight: 72.0 kDa) were purified via an amylose resin (GE Healthcare; running buffer: 20 mM Tris-HCl pH 7.5, 200 mM NaCl; elution buffer: 20 mM Tris-HCl, pH 7.5, 200 mM NaCl, 10 mM maltose). 10 µg of recombinant enzyme was loaded per lane. Proteins were stained with CBB after separation on a 7.5% SDS-PA gel. PageRuler Plus Prestained Protein Ladder (Thermo Fisher Scientific) was used as a protein molecular weight marker.

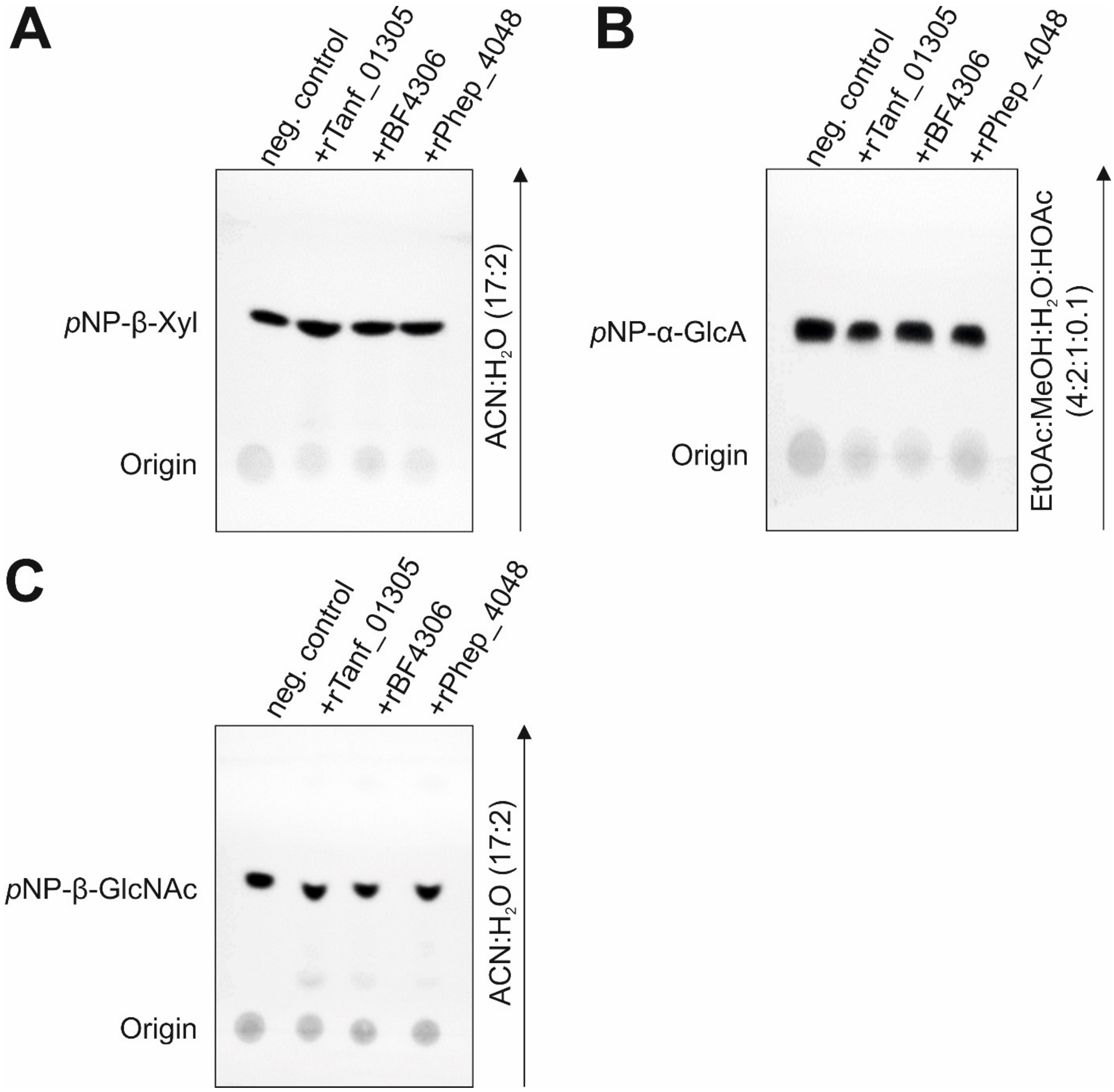

Figure S10. In vitro fucosyltransferase activity assays with other *p*NP-sugar substrates. Developed TLC plates show no formation of (**A**) *p*NP-*α*-D-Xyl-Fuc, (**B**) *p*NP-*α*-D-GlcA-Fuc or (**C**) *p*NP-β-D-GlcNAc-Fuc when using recombinant FucT from *T. forsythia* (rTanf_01305), *B. fragilis* (rBF4306) and *P. heparinus* (rPhep_4048), respectively.

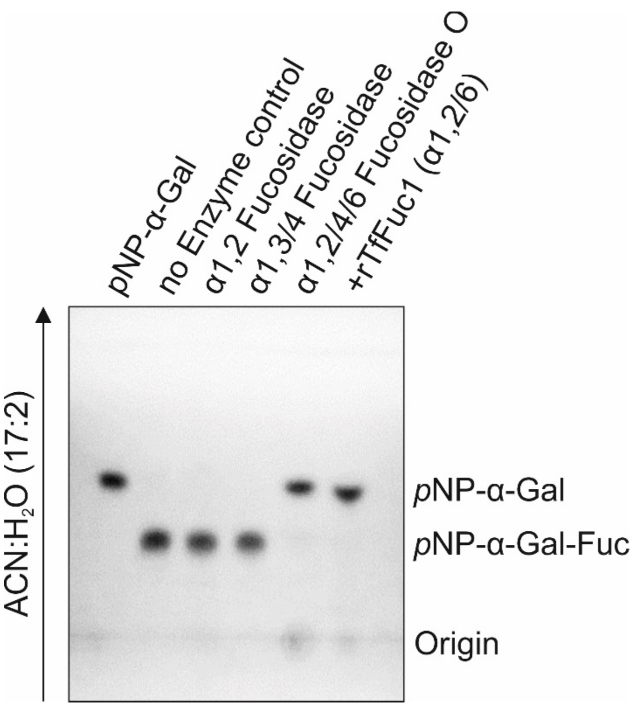

Figure S11. Fucosidase treatment of *p*NP-Gal-Fuc generated through Tanf_01305 activity on *p*NP-Gal. Commercially available fucosidases α1,2 fucosidase, α1,3/4 fucosidase and α1,2/4/6 fucosidase O as well as *T. forsythia* fucosidase rTfFuc1 were tested for their ability to cleave Fuc from *p*NP-α-Gal-Fuc. Reactions were analysed by TLC on silica gel 60 F_{254} plates. Fucosidase O and rTfFuc1 were active on *p*NP-α-Gal-Fuc.
